# Localized release of muscle-generated BDNF regulates the initial formation of postsynaptic apparatus at neuromuscular synapses

**DOI:** 10.1038/s41418-024-01404-4

**Published:** 2024-11-07

**Authors:** Jinkai Zhang, Hiu-Lam Rachel Kwan, Chi Bun Chan, Chi Wai Lee

**Affiliations:** 1 Department of Biology, Faculty of Science, Hong Kong Baptist University, Hong Kong, China; 2 School of Biomedical Sciences, LKS Faculty of Medicine, The University of Hong Kong, Hong Kong, China; 3 School of Biological Sciences, Faculty of Science, The University of Hong Kong, Hong Kong, China; 4 Golden Meditech Centre for NeuroRegeneration Sciences, Hong Kong Baptist University, Hong Kong, China

**Keywords:** Somatic system, Extracellular matrix

## Abstract

Growing evidence indicates that brain-derived neurotrophic factor (BDNF) is produced in contracting skeletal muscles and is secreted as a myokine that plays an important role in muscle metabolism. However, the involvement of muscle-generated BDNF and the regulation of its vesicular trafficking, localization, proteolytic processing, and spatially restricted release during the development of vertebrate neuromuscular junctions (NMJs) remain largely unknown. In this study, we first reported that BDNF is spatially associated with the actin-rich core domain of podosome-like structures (PLSs) at topologically complex acetylcholine receptor (AChR) clusters in cultured *Xenopus* muscle cells. The release of spatially localized BDNF is tightly controlled by activity-regulated mechanisms in a calcium-dependent manner. Live-cell time-lapse imaging further showed that BDNF-containing vesicles are transported to and captured at PLSs in both aneural and synaptic AChR clusters for spatially restricted release. Functionally, BDNF knockdown or furin-mediated endoproteolytic activity inhibition significantly suppresses aneural AChR cluster formation, which in turn affects synaptic AChR clustering induced by nerve innervation or agrin-coated beads. Lastly, skeletal muscle-specific BDNF knockout (MBKO) mice exhibit structural defects in the formation of aneural AChR clusters and their subsequent recruitment to nerve-induced synaptic AChR clusters during the initial stages of NMJ development in vivo. Together, this study demonstrated the regulatory roles of PLSs in the intracellular trafficking, spatial localization, and activity-dependent release of BDNF in muscle cells and revealed the involvement of muscle-generated BDNF and its proteolytic conversion in regulating the initial formation of aneural and synaptic AChR clusters during early NMJ development in vitro and in vivo.

## Introduction

During the nervous system development, individual neurons sense a variety of different soluble proteins that profoundly influence their survival, outgrowth, and differentiation. The role of target-derived trophic factors, neurotrophins, has been well recognized in these processes [1, 2]. Skeletal muscles, the synaptic targets of motor neurons, are one of the major sources of several neurotrophins, including brain-derived neurotrophic factor (BDNF), neurotrophin-3 (NT-3), and neurotrophin-4/5 (NT-4/5) [3, 4]. Among them, BDNF is the most extensively studied member and is well-known for its profound effects on the structure and function of both central and peripheral nervous systems [5–7]. At the vertebrate neuromuscular junctions (NMJs), BDNF is generally known to enhance neurotransmitter release during synaptic plasticity [8–12]. However, a previous study indicated that recombinant BDNF treatment not only promotes spinal neuron survival and outgrowth, but also suppresses agrin synthesis and deposition in presynaptic neurons, leading to the inhibition of synapse formation in nerve-muscle cocultures [13]. Apart from the retrograde functions of BDNF in the presynaptic neurons, bath application of exogenous BDNF is also known to modulate agrin-induced postsynaptic differentiation in cultured myotubes [14]. However, whether localized release of endogenously expressed BDNF at the physiological level regulates postsynaptic differentiation in skeletal muscles remains unknown.

Like other neurotrophins, BDNF is first synthesized as a pre-proBDNF in the endoplasmic reticulum, where its pre-sequence is immediately cleaved off to produce the 32 kDa precursor proBDNF [15]. proBDNF is then transported to the Golgi apparatus for sorting into either constitutive or regulated secretory vesicles [16]. This precursor form can be activated through proteolytic cleavage at the consensus sequence (R-X-K/R-R) of the C-terminus to generate the 14 kDa mature form mBDNF by the action of either furin and convertases intracellularly or matrix metalloproteinases (MMPs) and plasmin extracellularly [17, 18]. Intriguingly, proBDNF and mBDNF preferentially activate the p75 neurotrophin receptor (p75^NTR^) and tropomyosin-related kinase B (TrkB), respectively, which often result in opposite cellular responses [19, 20]. For example, mBDNF promotes the stabilization of active terminals, while proBDNF causes the elimination of inactive terminals, during synaptic competition and elimination at polyinnervated NMJs in vivo [21, 22], indicating the physiological importance of the proteolytic conversion of BDNF in determining the cellular responses. However, the molecular mechanisms underlying the subcellular localization, spatially restricted release, and proteolytic conversion of BDNF in skeletal muscles warren further investigations.

Previous studies from our laboratory and others have shown that topologically complex postsynaptic apparatus can spontaneously be formed in *Xenopus* primary muscle cells, immortalized C2C12 myotubes, and human primary myotubes cultured on extracellular matrix (ECM)-coated substratum [23–25]. These ECM-induced aneural AChR clusters exhibit progressive topological transformations similar to those observed during the structural maturation of postsynaptic apparatus at postnatal NMJs in vivo [26]. Dynamic actin-rich adhesive organelles, podosome-like structures (PLSs), are usually detected at the AChR-poor regions of those postsynaptic apparatus [23, 27–29], which can direct vesicular trafficking and surface targeting of membrane-type 1 matrix metalloproteinase (MT1-MMP) to focally mediate ECM remodeling during topological maturation and junctional fold development at the postsynaptic apparatus [24, 30]. In the present study, we further showed that endogenous BDNF is spatially enriched at the actin-rich core domain of PLSs within aneural AChR clusters in cultured *Xenopus* muscle cells, and its secretion is tightly controlled by activity-regulated and calcium-dependent mechanisms. Next, live-cell time-lapse imaging further showed the intracellular transport of BDNF-containing vesicles to PLSs at aneural and synaptic AChR clusters for spatially restricted release. Reduced expression of endogenous BDNF proteins by antisense morpholino oligonucleotide (MO) or pharmacological inhibition of furin-mediated proteolytic conversion from proBDNF to mBDNF significantly suppressed the formation of aneural AChR clusters in cultured muscle cells, which in turn inhibited synaptic AChR clustering induced by agrin beads or nerve innervation. Finally, in skeletal muscle-specific BDNF knockout (MBKO) mouse model, we showed that BDNF is involved in the initial formation of aneural AChR clusters and their subsequent recruitment to nerve-induced synaptic AChR clusters during the early stages of NMJ development in vivo. Taken together, the results of this study demonstrated the regulatory roles of actin-dependent PLSs in the spatial localization and activity-dependent release of BDNF in muscle cells and revealed a previously unappreciated role of muscle BDNF in regulating the initial formation of prepatterned AChR clusters and their subsequent contribution to the formation and topological remodeling of the nerve-induced postsynaptic apparatus at developing NMJs in vitro and in vivo.

## Results

### Muscle-generated BDNF and TrkB receptors are spatially enriched in aneural AChR clusters

**Fig. 1:**
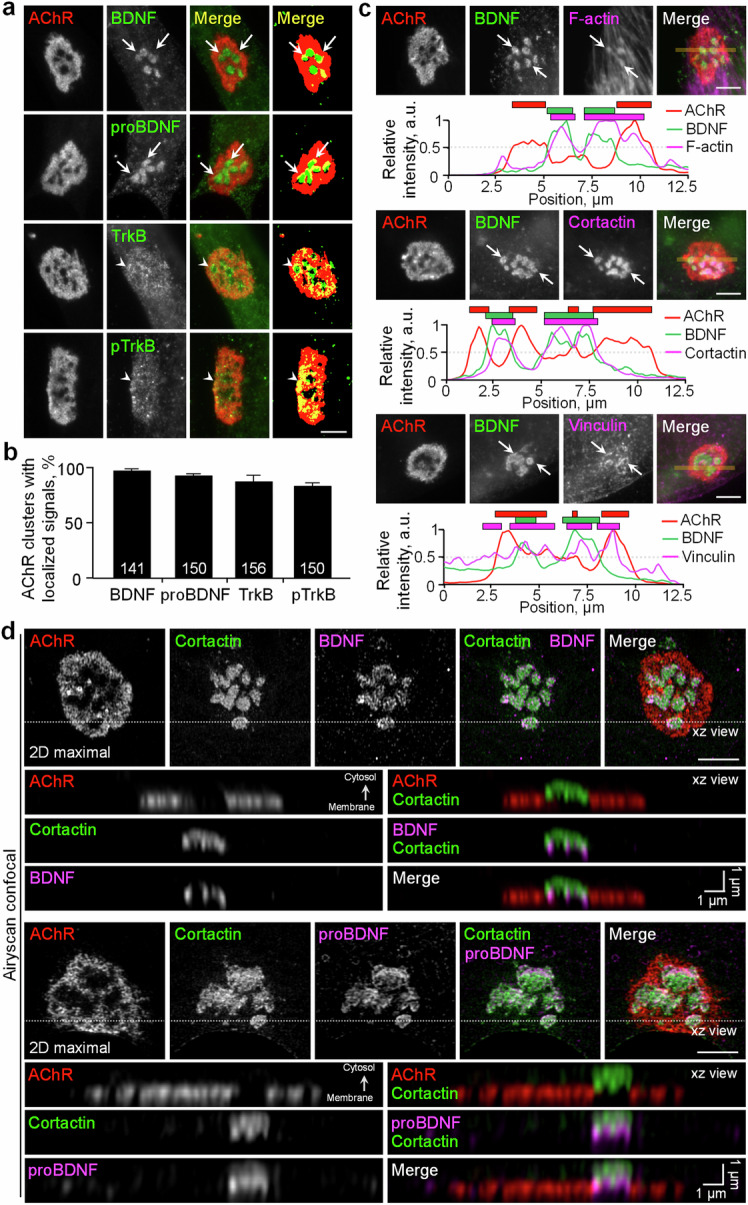
Endogenous BDNF and TrkB receptors are spatially associated with PLS core regions in aneural AChR clusters. Representative images (**a**) and quantification (**b**) showing the spatial localization of endogenous BDNF, proBDNF, TrkB, and pTrkB receptors at aneural AChR clusters in cultured *Xenopus* muscles. Arrows and arrowheads indicate localized signals at the perforated and AChR-rich regions of aneural clusters, respectively. **c** Representative images showing the spatial association (arrows) between BDNF and PLS at perforated aneural AChR clusters. The yellow lines indicate the region-of-interest for generating line profiles. Line profile plots showing the relative fluorescence intensities of AChR (red), BDNF (green), and PLS core and cortex markers (magenta). The colored boxes indicate the spatial enrichment of markers with their respective fluorescence signals above the cutoff intensity of 50%. **d** Maximal projection of Airyscan confocal z-stack images showing the spatial enrichment of BDNF (top panels) or proBDNF (bottom panels) at PLS core regions within aneural AChR clusters. The orthogonal xz views show the differential distributions of AChR, cortactin, and BDNF/proBDNF. Scale bars represent 5 µm, unless stated otherwise.

To examine the subcellular localization of BDNF and its precursor proteins in cultured muscle cells, we performed immunostaining and detected the spatial enrichment of endogenous BDNF and proBDNF at the perforated sites of aneural AChR clusters in *Xenopus* primary muscle cells cultured on ECM-coated substrates (Fig. 1a, b). The immunostaining signals could be completely abolished after pre-incubating the primary antibodies with their corresponding immunogen peptide only but not vice versa, validating the specificity of antibodies in recognizing BDNF and proBDNF proteins differentially (Fig. S1a). As BDNF binds to and activates a highly homologous receptor tyrosine kinase known as TrkB receptor, we next investigated the localization of total and active TrkB receptors at aneural AChR clusters. Intriguingly, immunostaining experiments showed that TrkB and phosphorylated active TrkB (pTrkB) were preferentially concentrated in AChR-rich regions of the clusters (Fig. 1a, b).

As the perforated sites of AChR clusters contain PLSs that actively participate in the topological maturation of the postsynaptic apparatus via MT1-MMP-mediated ECM remodeling [24, 31], we next examined whether the spatially enriched BDNF proteins are colocalized with PLSs at AChR clusters. The location of PLSs was identified by probing filamentous actin (F-actin) with fluorescent phalloidin, the actin-rich core domain with cortactin antibody, and the cortex domain with vinculin antibody. Here, we detected endogenous BDNF proteins that were highly colocalized with the core domain of PLSs (Fig. 1c), as clearly reflected in the line profile analyses. To further determine the precise localization of BDNF and proBDNF in relation to the PLSs, we then performed Airyscan confocal super-resolution microscopy. Consistent with the data above, the maximal projection of z-stack images showed that BDNF and proBDNF were highly co-localized with cortactin at the perforated sites of aneural AChR clusters (Fig. 1d). Interestingly, orthogonal views of the reconstructed 3-dimensional Airyscan confocal images revealed slight differences in their spatial localization, in which both BDNF and proBDNF were preferentially enriched at the base of cortactin-labeled PLS core domain. These data suggested that PLS is likely involved in the spatial localization of BDNF and proBDNF at aneural AChR clusters in muscle cells.

**Fig. 2:**
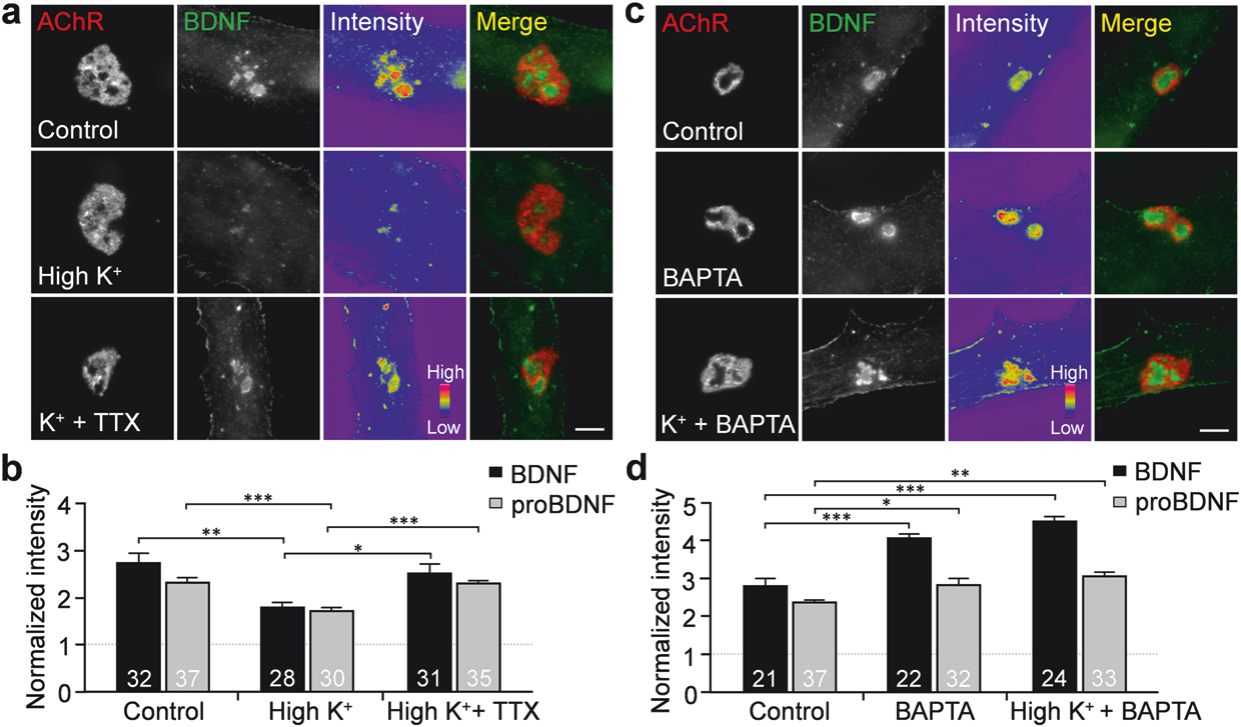
Constitutive and activity-regulated release of BDNF at aneural AChR clusters are regulated by calcium-dependent pathways. Representative images (**a**) and quantification (**b**) showing that high potassium stimulation greatly reduced the spatially enriched endogenous BDNF at aneural AChR clusters. The depolarization-induced release of BDNF was significantly suppressed by TTX co-treatment. Representative images (**c**) and quantification (**d**) showing the requirement of intracellular calcium ions for both constitutive and activity-regulated release of BDNF at aneural AChR clusters. Scale bars represent 5 μm. 8-bit pseudo-color images highlight the relative fluorescence intensity. Data are mean ± SEM. The numbers indicated in the bar regions represent the total numbers of muscle cells quantified from 3 independent experiments. *, **, *** represent *p* ≤ 0.05, 0.01, and 0.001, respectively (one-way ANOVA with Dunnett’s multiple comparisons test).

To investigate whether the spatially localized BDNF and proBDNF proteins in aneural AChR clusters are releasable in response to stimulation, we briefly stimulated the muscle cells with a high potassium (50 mM) solution for 5 min, immediately followed by cell fixation and immunostaining. We found that the fluorescence intensities of both intracellular BDNF and proBDNF in aneural AChR clusters were significantly reduced upon high potassium stimulation (Fig. 2a, b), demonstrating the activity-dependent release of intracellular BDNF and proBDNF to the extracellular environment. Next, we used tetrodotoxin (TTX), a potent voltage-gated sodium channel blocker, to inhibit the generation of action potentials. TTX treatment completely abolished the reduction in BDNF and proBDNF signals induced by high potassium stimulation. As calcium is known to be an important regulator of the exocytosis of secretory vesicles [32], we thus used the cell-permeable intracellular calcium chelator BAPTA-AM to examine the requirement of calcium ions for the release of BDNF and proBDNF proteins at aneural AChR clusters. In the presence of BAPTA-AM, we observed a significant increase in the intensity of BDNF and proBDNF signals (Fig. 2c, d) at the perforated regions of aneural AChR clusters. In addition, BAPTA-AM treatment completely abolished the reduction in BDNF and proBDNF signals induced by high potassium stimulation. These results indicated the essential roles of calcium influx in both spontaneous and activity-dependent release of spatially localized BDNF and proBDNF at aneural AChR clusters.

### Actin dynamics is required for the spatial localization of BDNF at aneural AChR clusters

**Fig. 3:**
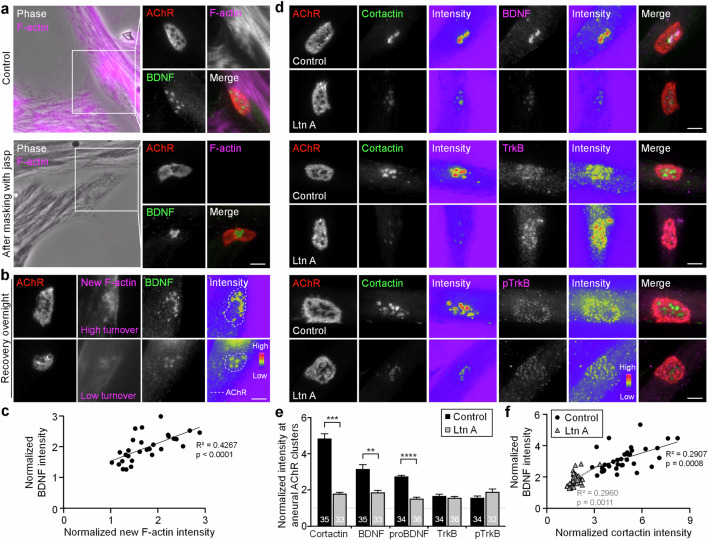
Actin dynamics is required for the spatial localization of BDNF at aneural AChR clusters. **a** Representative images showing the complete masking of stable F-actin pools at sarcomeres in the entire muscle cells after 3-h jasplakinolide (jasp) treatment. Representative images (**b**) showing the enrichment of newly synthesized F-actin at perforated aneural AChR clusters in muscle cells post-treated with jasplakinolide after overnight recovery. A scatter plot analysis (**c**) indicating a positive correlation between the newly synthesized F-actin and BDNF fluorescence intensity at perforated aneural AChR clusters. *n* = 29 from 3 independent experiments. **d**–**f** Representative images (**d**) and quantification (**e**) showing that actin assembly inhibition by latrunculin A (Ltn A) greatly reduced the spatial localization of cortactin and BDNF, but not TrkB and pTrkB signals, in aneural AChR clusters. Cortactin and BDNF signals at AChR cluster perforations, and TrkB and pTrkB signals at the entire AChR cluster regions, were normalized to the in-cell background. A scatter plot analysis (**f**) indicating a positive correlation between cortactin and BDNF fluorescence intensity at perforated aneural AChR clusters in control and Ltn A-treated muscle cells. *n* = 35 (Control) and 33 (Ltn A) muscle cells from 3 independent experiments. Scale bars represent 5 μm. 8-bit pseudo-color images highlight the relative fluorescence intensity. Data are mean ± SEM. The numbers indicated in the bar regions represent the total number of muscle cells quantified from 3 independent experiments. **, *** represent *p* ≤ 0.01, and 0.001 respectively (Student’s *t* test).

As BDNF is spatially localized at the actin-rich core domain of PLSs, we next investigated the requirement of actin dynamics for the spatial localization of BDNF at aneural AChR clusters. In control muscle cells, BDNF proteins were colocalized with fluorescent phalloidin-labeled actin-rich PLS core domains at AChR clusters (Fig. 3a). To probe the newly synthesized F-actin, cultured muscle cells were first treated with 2.5 µM jasplakinolide that masks the pre-existing F-actin at myofibril structures in the entire cell in accordance with the established procedures [33], after which no signals could be detected by fluorescent phalloidin staining immediately after jasplakinolide masking. Interestingly, after overnight recovery, fluorescent phalloidin signals representing newly synthesized F-actin were concentrated at the perforated sites of aneural AChR clusters, where BDNF proteins were also found to be concentrated (Fig. 3b). By analyzing a pool of aneural AChR clusters in muscle cells (*n* = 29) from three independent experiments, we detected a positive correlation between BDNF and newly synthesized F-actin signals in the regions of aneural AChR clusters (Fig. 3c), indicating the possible requirement of actin dynamics for the spatial localization of BDNF at topologically complex postsynaptic apparatus.

To further determine whether actin polymerization is required for BDNF localization, we used the actin drug latrunculin A (Ltn A) to sequester actin monomers and thereby inhibit actin polymerization [34]. Since actin polymerization is required for the formation of AChR clusters and the spreading of cultured muscle cells [33, 35], Ltn A was added to 3-day-old muscle cells after the formation of aneural AChR clusters, followed by immunostaining for cortactin, an actin-binding protein that is involved in different stages of PLS formation [36]. In cultured cells treated with 10 µM Ltn A for 4 h, we detected a significant reduction in the immunostaining signals of BDNF, proBDNF, and cortactin at aneural AChR clusters (Fig. 3d, e). On the other hand, no changes in TrkB or pTrkB signals were observed between the control and Ltn A-treated muscle cells. Consistent with the data above showing a positive correlation between BDNF and newly polymerized F-actin signals (Fig. 3c), we also detected a positive correlation between BDNF and cortactin signals at aneural AChR clusters in control and Ltn A-treated muscle cells (Fig. 3f). As our previous study demonstrated the involvement of actin-based structures in regulating the vesicular trafficking and surface insertion of newly synthesized AChRs [23], here we also showed that Ltn A treatment significantly reduced not only the spatially localized BDNF signals, but also the newly inserted AChR signals, at aneural AChR clusters (Fig. S2). Taken together, these data suggest that dynamic actin turnover at the PLS core domain is required for the spatial localization of BDNF and AChR molecules at topologically complex postsynaptic apparatus.

### Vesicular trafficking and localized release of BDNF are directed by PLSs at aneural and synaptic AChR clusters

**Fig. 4:**
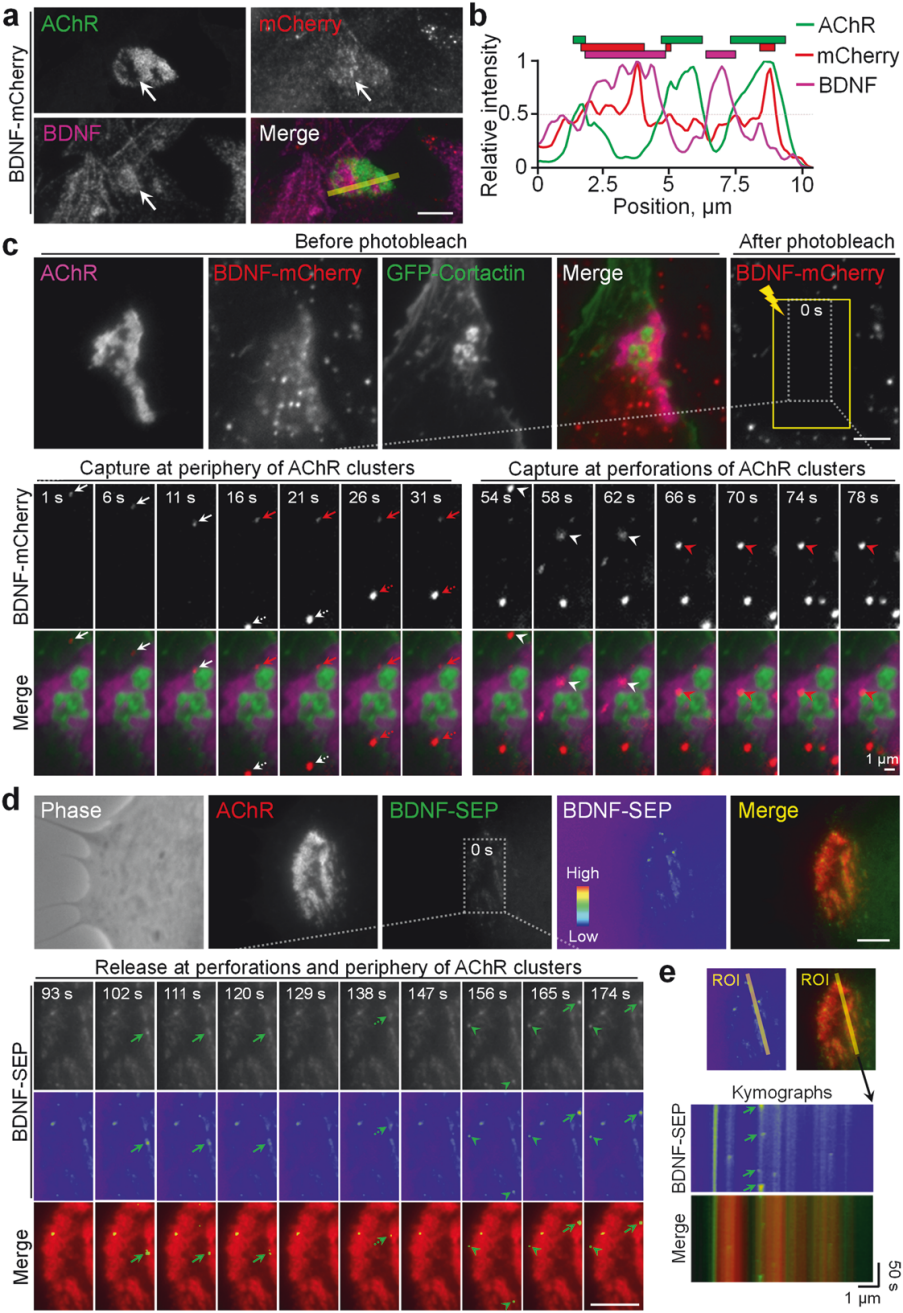
BDNF vesicles are transported to aneural AChR clusters for spatially restricted release. Representative images (**a**) and line profile analyses (**b**) showing BDNF immunostaining on BDNF-mCherry-expressing muscle cells. BDNF-mCherry signals were found to be partially colocalized with endogenous BDNF proteins at aneural AChR clusters (arrows). The yellow line indicates the region-of-interest for generating line profiles. The colored regions indicate the respective fluorescence signals above the cutoff intensity of 50%. **c** Representative TIRF-FRAP images showing the intracellular trafficking and local capturing of BDNF vesicles at aneural AChR clusters in BDNF-mCherry-expressing muscle cells. A time-lapse series of a region (dotted box) showed the local capture (red arrows and arrowheads) of multiple moving BDNF-mCherry signals (white arrows and arrowheads) at the periphery (left panels) or perforations (right panels) of AChR clusters. The yellow box represents the photobleached region. **d**, **e** Representative time-lapse images showing localized BDNF release at aneural AChR clusters in BDNF-SEP-expressing muscle cells. A time-lapse series (**d**) and kymographs (**e**) constructed along the yellow lines indicating multiple events of BDNF-SEP release (arrows and arrowheads) at aneural AChR clusters. 8-bit pseudo-color images highlight the change in the relative intensity of BDNF-SEP signals upon spatially restricted release. Scale bars represent 5 μm, unless stated otherwise.

Our recent study showed that vesicular trafficking and surface insertion of MT1-MMP are directed to PLSs for mediating focal ECM degradation required of AChR cluster remodeling and redistribution [24]. To further investigate the spatiotemporal regulation of BDNF trafficking and localization in live cells, we overexpressed a plasmid encoding BDNF tagged with the red fluorescent protein mCherry (BDNF-mCherry) in cultured *Xenopus* muscle cells at low expression levels to minimize the possible effects of overexpression artifacts. In BDNF-mCherry-expressing muscle cells, we detected spatially localized BDNF immunostaining signals at aneural AChR clusters as observed in the control cells above, and some mCherry signals were colocalized with BDNF immunostaining signals (Fig. 4a, b), indicating the feasibility of using the BDNF-mCherry construct to study the vesicular trafficking of BDNF in live cells without noticeable changes in the subcellular localization of endogenous BDNF. To demonstrate the intracellular transport and local capture of BDNF-mCherry vesicles at the PLSs, we performed total internal reflection fluorescence (TIRF) microscopy in combination with fluorescence recovery after photobleaching (FRAP) experiments on muscle cells expressing both BDNF-mCherry and GFP-cortactin, a PLS core marker. Before photobleaching, some BDNF-mCherry signals could be detected in the vicinity of aneural AChR clusters in this time-lapse series (Fig. 4c). After photobleaching, we detected several events of dynamic BDNF-mCherry vesicular trafficking (white arrows and arrowheads), followed by local capturing (red arrows and arrowheads) at either the periphery (at 16 s and 26 s) or the perforations (at 66 s) of AChR clusters. Specifically, local capturing of BDNF-mCherry vesicles was detected at sites where GFP-cortactin was localized at AChR-poor perforated sites, suggesting that cortactin-labeled PLSs may direct the intracellular trafficking and capture of BDNF vesicles at AChR clusters in cultured muscles. It is noteworthy that in cultured muscle cells with low expression of BDNF-mCherry, we detected a slight but significant increase in the intensity of AChR clusters compared to those in wild-type muscle cells (Fig. S3). This finding further supported the potential roles of BDNF in regulating AChR cluster formation.

To further investigate whether these locally captured BDNF vesicles can be released at aneural AChR clusters, we used another plasmid encoding BDNF tagged with the pH-sensitive probe super-ecliptic pHluorin (BDNF-SEP) [37]. The fluorescence signal of BDNF-SEP is quenched inside the acidic vesicular compartment but drastically increases at natural pH upon releasing into the extracellular environment. In BDNF-SEP-expressing muscle cells, time-lapse imaging revealed that fluorescence bursts of BDNF-SEP signals (green arrows and arrowheads), indicating separate events of spatially restricted release of BDNF vesicles, were also detected at the perforations (at 102 s, 138 s, and 165 s) and periphery (at 156 s) of AChR clusters in cultured muscle cells (Fig. 4d). Kymograph analyses further revealed multiple events of BDNF release that occurred at the same sites within AChR clusters over the 180 s time-lapse imaging period (Fig. 4e). Taken together, in addition to the vesicular trafficking and surface targeting of membrane proteins, such as MT1-MMP and AChR, as shown in our previous studies [23, 24], we here further showed that PLSs also direct the vesicular trafficking and localized release of secretory BDNF proteins at aneural AChR clusters in muscle cells.

**Fig. 5:**
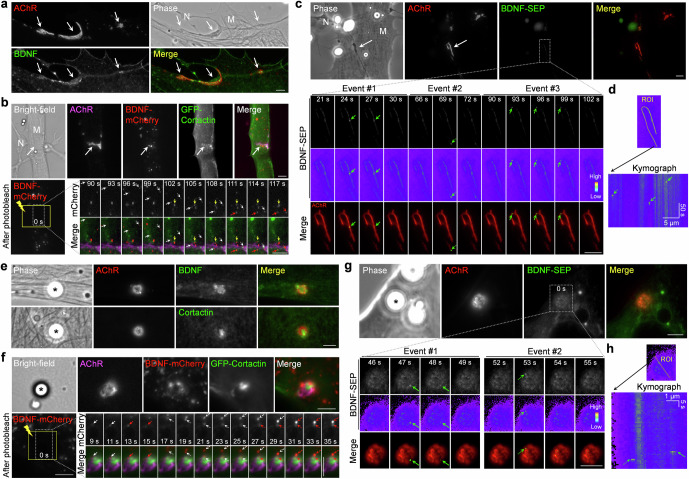
BDNF vesicles are transported to and released at synaptic AChR clusters induced by spinal neurons or agrin beads. **a** Representative images showing the spatial enrichment of endogenous BDNF proteins at nerve-induced AChR clusters (arrows). M and N indicate muscle and spinal neuron, respectively. **b** Representative TIRF-FRAP images showing the intracellular trafficking and local capturing of BDNF-mCherry vesicles at the nerve-induced AChR clusters (arrows). Bottom panels: After photobleaching, time-lapse montage images show that BDNF-mCherry vesicles were dynamically transported to (white arrows) and locally captured at (red arrows) the sites of nerve-induced AChR clusters. Yellow arrows indicate an example of immobile BDNF-mCherry vesicles. **c**, **d** Representative time-lapse images (**c**) showing the spatial release of BDNF-SEP proteins at nerve-induced AChR clusters. White arrows indicate synaptic AChR clusters at nerve-muscle contacts. Bottom panels: Green arrows indicate the spatial release of BDNF-SEP proteins in association with synaptic AChR clusters at the nerve-muscle contacts. A kymograph (**d**) was constructed along the indicated yellow line that shows the spatiotemporal release of BDNF-SEP proteins at nerve-induced AChR clusters. Green arrows indicate three vesicular BDNF-SEP release events (in **c**) along the length of nerve-induced AChR clusters over a period of 180 s. **e** Representative images showing the spatial localization of endogenous BDNF and cortactin at agrin bead-induced AChR clusters. **f** Representative TIRF-FRAP images showing the local capturing of BDNF-mCherry vesicles at agrin bead-induced AChR clusters. After photobleaching, time-lapse montage images show that BDNF-mCherry vesicles were transported to (white arrows) and captured at (red arrows) the sites of agrin bead-induced AChR clusters. **g**, **h** Representative time-lapse images (**g**) showing the spatial release of BDNF-SEP proteins (green arrows) at agrin bead-induced AChR clusters. A kymograph (**h**) constructed along the indicated yellow line showing the spatiotemporal release of BDNF-SEP proteins at agrin bead-induced AChR clusters. Green arrows indicate two vesicular BDNF-SEP release events (in **g**) at bead-induced AChR clusters over a period of 180 s. Scale bars represent 5 μm, unless stated otherwise. Asterisks indicate bead-muscle contacts. 8-bit pseudo-color images highlight the change in the relative intensity. Indicated time stamps represent the elapsed time after photobleaching (in **b**, **d**) or the start of time-lapse recording (in **c**, **g**).

Next, we investigated whether the trafficking, localization, and release of muscle-generated BDNF also occur at synaptic AChR clusters induced by spinal neurons. By performing BDNF immunostaining in 3-day-old *Xenopus* nerve-muscle co-cultures, we detected spatial enrichment of endogenous BDNF proteins to be colocalized with synaptic AChR clusters along the nerve-contacted trails in muscle cells (Fig. 5a). Then, we adopted similar approaches as described above involving the use of BDNF-mCherry to visualize the intracellular trafficking and local capturing of BDNF vesicles (Fig. 5b), followed by BDNF-SEP to detect the spatially restricted BDNF release (Fig. 5c), at nerve-muscle contact sites. Specifically, we performed TIRF-FRAP experiments on chimeric co-cultures composed of BDNF-mCherry-expressing muscle cells and wild-type neurons. After photobleaching, we detected the dynamic movement (white arrows) of BDNF-mCherry vesicles into the photobleached region, followed by the immobilization (red arrows; at 111 s and 117 s) of signals at nerve-induced AChR clusters in the postsynaptic muscle cells (Fig. 5b). In this time-lapse recording of chimeric co-cultures composed of BDNF-SEP-expressing muscle cells and wild-type neurons, we detected fluorescence bursts of BDNF-SEP signals (green arrows; at 24 s, 69 s, and 93 s) along the nerve-muscle contacts (Fig. 5c). Kymograph analyses further revealed several events of transient fluorescence bursts in this time-lapse recording (Fig. 5d), representing the localized release of diffusible muscle BDNF proteins at nerve-induced postsynaptic apparatus.

To further demonstrate the vesicular trafficking and localized release of muscle BDNF at synaptic AChR clusters, agrin beads were added on cultured muscle cells to induce postsynaptic differentiation in a spatiotemporally controllable manner [23]. After 24-h agrin bead stimulation, immunostaining experiments showed that endogenous BDNF, cortactin, and AChR clusters were all concentrated at the bead-muscle contacts (Fig. 5e). In live muscle cells expressing BDNF-mCherry, we detected the dynamic movement (white arrows) of BDNF-mCherry vesicles into the photobleached region, followed by immobilization (red arrows; at 13 s, 27 s, and 31 s) of signals at agrin bead-induced AChR clusters (Fig. 5f), consistent with the data on synaptic AChR clusters induced by nerve innervation (Fig. 5b). Similarly, time-lapse imaging of BDNF-SEP-expressing muscle cells also showed fluorescence bursts of BDNF-SEP signals (green arrows; at 47 s and 53 s) in this agrin bead-muscle contact (Fig. 5g), which could be clearly seen in the kymograph (Fig. 5h). Taken together, our results demonstrated that BDNF vesicles are targeted to both ECM-induced aneural AChR clusters and nerve-/agrin-induced synaptic AChR clusters for localized release from muscle cells.

### Muscle BDNF expression and its proteolytic conversion are required for aneural AChR cluster formation

**Fig. 6:**
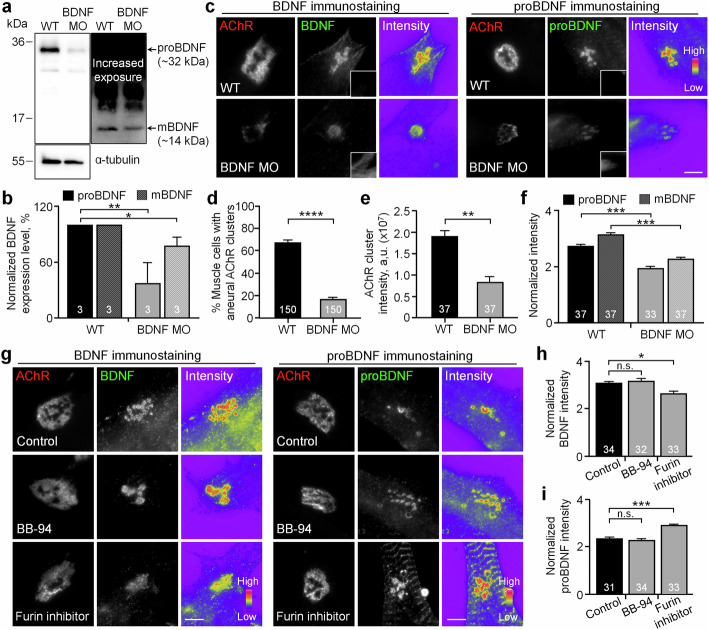
BDNF knockdown or furin inhibition affects aneural AChR cluster formation in cultured muscle cells. Western blot analysis (**a**) and quantification (**b**) showing the effective knockdown of both endogenous BDNF and proBDNF expression by antisense BDNF MO. Anti-α-tubulin was used as a loading control. **c–f** Representative images (**c**) showing the reduced intensity of aneural AChR clusters, BDNF, and proBDNF signals in BDNF MO muscle cells. Insets: Fluorescent dextran signals indicate the presence of the MO in muscle cells. Quantification showing the effects of BDNF knockdown on the formation of aneural AChR clusters (**d**) and their normalized fluorescence intensity (**e**), as well as the normalized intensity of BDNF and proBDNF (**f**) at the perforated regions of aneural AChR clusters. **g**–**i** Representative images (**g**) showing the effects of BB-94 or furin inhibitor on the localization of BDNF and proBDNF at perforated aneural AChR clusters. Quantification showing the effects of BB-94 or furin inhibitor on the normalized intensity of BDNF (**h**) and proBDNF (**i**) at the perforated regions of aneural AChR clusters. Scale bars represent 5 μm. 8-bit pseudo-color images highlight the relative fluorescence intensity. Data are means ± SEM. The numbers indicated in the bar regions represent the total numbers of blots (**b**) or muscle cells (**d**–**f**, **h**, **i**) measured from three independent experiments. *, **, ***, **** represent *p* ≤ 0.05, 0.01, 0.001, and 0.0001, respectively (Student’s *t* test (**b**, **d**–**f**) or one-way ANOVA with Tukey’s multiple comparisons test (**h**, **i**)).

To investigate the requirement of muscle BDNF for AChR clustering, we used antisense MOs to knock down endogenous BDNF expression in *Xenopus* embryos. In wild-type embryos, Western blot data showed that proBDNF (~32 kDa) was relatively higher abundant than mBDNF (~14 kDa) in the myotomal tissue lysates (Fig. 6a). In BDNF MO embryos, we detected a significant reduction in both proBDNF and mBDNF expression (Fig. 6b). Next, we examined the effects of BDNF MO on the formation of aneural AChR clusters. We found that the percentage of BDNF MO muscle cells with aneural AChR clusters was significantly reduced (Fig. 6d). Among those aneural AChR clusters that were detected in BDNF MO muscle cells, immunostaining experiments also showed a significant reduction in the fluorescence intensities of AChR and proBDNF signals (Fig. 6c, e, f). Despite the fact that mBDNF expression is relatively less abundant in the myotomal tissue lysate, the spatially enriched BDNF signals at aneural AChR clusters were significantly reduced in BDNF MO muscle cells. Taken together, our findings indicate that reduced endogenous expression and altered subcellular localization of muscle BDNF (proBDNF and mBDNF) inhibit the initial formation of aneural AChR clusters in cultured muscle cells.

The proteolytic conversion of proBDNF to mBDNF is known to be regulated either intracellularly by furin/proprotein convertases or extracellularly by plasmin/MMPs [38]. Therefore, we investigated the effects of the broad-spectrum MMP inhibitor BB-94 and furin inhibitor on the spatial localization of BDNF and proBDNF at aneural AChR clusters. Interestingly, no significant difference in BDNF or proBDNF intensity was detected between the control and 5 μM BB-94-treated muscle cells (Fig. 6g–i). In contrast, 10 μM furin inhibitor caused a significant reduction in BDNF intensity, together with a significant increase in proBDNF intensity, at aneural AChR clusters, indicating that the proteolytic conversion of proBDNF to mBDNF is primarily mediated by furin intracellularly in cultured muscle cells.

### Muscle BDNF expression and its proteolytic conversion are required for synaptic AChR cluster formation

**Fig. 7:**
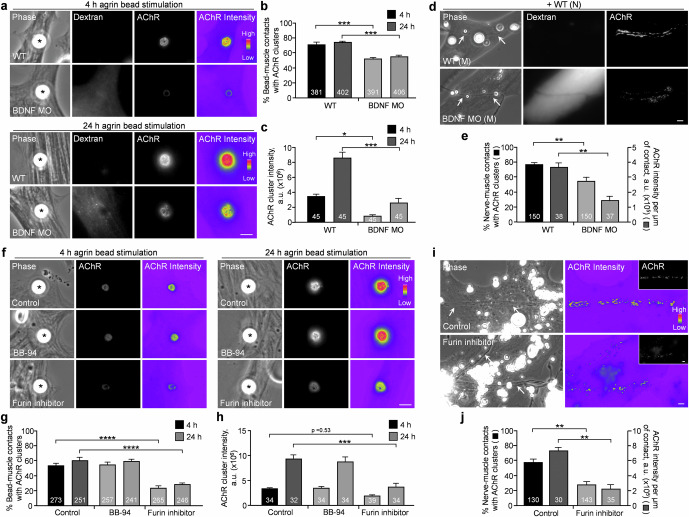
BDNF knockdown or furin inhibition affects synaptic AChR cluster formation induced by spinal neurons or agrin beads. Representative images (**a**) and quantification showing the effects of BDNF knockdown on the formation of agrin bead-induced AChR clusters (**b**) and their normalized fluorescence intensity (**c**) after 4- or 24-h stimulation. Representative images (**d**) and quantification (**e**) showing the effects of muscle BDNF knockdown on nerve-induced AChR clustering and their normalized fluorescence intensity per unit length of nerve-muscle contacts. Arrows indicate nerve-muscle contacts. Representative images (**f**) and quantification showing the effects of BB-94 or furin inhibitor on the formation of agrin bead-induced AChR clusters (**g**) and their normalized fluorescence intensity at bead-muscle contacts (**h**). Representative images (**i**) and quantification (**j**) showing the effects of furin inhibitor treatment on nerve-induced AChR clustering and their normalized fluorescence intensity per unit length of nerve-muscle contacts. Arrows indicate nerve-muscle contacts. Scale bars represent 5 μm. Asterisks indicate bead-muscle contacts. Fluorescent dextran signals indicate the presence of BDNF MO in muscle cells. 8-bit pseudo-color images highlight the relative fluorescence intensity. Data are means ± SEM. The numbers indicated in the bar regions represent the total numbers of bead-muscle contacts (**b**, **c**, **g**, **h**) or nerve-muscle contacts (**e**, **j**) measured from three independent experiments. *, **, ***, **** represent *p* ≤ 0.05, 0.01, 0.001, and 0.0001, respectively (one-way ANOVA with Tukey’s multiple comparisons test (**b**, **c**, **g**, **h**), and Student’s *t* test (**e**, **j**)).

Upon synaptogenic stimulation, aneural AChR clusters are redistributed to the nascent synaptic sites for the assembly of postsynaptic apparatus [39–42]. As BDNF knockdown greatly inhibited the formation of aneural AChR clusters (Fig. 6d), we next investigated the effects of BDNF MO on synaptic AChR clustering in cultured muscle cells induced by either local application of agrin beads or coculture with spinal neurons. In wild-type muscle cells, agrin beads effectively induced AChR clustering at the bead-muscle contacts in a time-dependent manner (Fig. 7a), with a progressive increase in AChR intensity from 4 to 24 h after agrin bead stimulation. In BDNF MO muscle cells, we found that the percentage of bead-muscle contacts with AChR clustering and their fluorescence intensity were significantly lower than those in the control cells at the same timepoints (Fig. 7b, c). Consistent with these findings, chimeric co-cultures composed of BDNF MO muscle cells and wild-type neurons also exhibited a significant inhibition in the formation of synaptic AChR clusters at nerve-muscle contacts (Fig. 7d, e).

Next, we examined the involvement of proteolytic conversion of proBDNF to mBDNF in synaptic AChR clustering. We found that furin inhibitor treatment strongly suppressed the formation of synaptic AChR clusters induced by either agrin bead stimulation (Fig. 7f–h) or nerve innervation (Fig. 7i, j), consistent with the effects on aneural AChR clusters (Fig. 6g–i). In contrast, agrin bead-induced AChR clustering was not affected by the MMP inhibitor BB-94 (Fig. 7f–h). Although our previous study showed that BB-94 treatment suppresses synaptic AChR clustering in the nerve-muscle co-cultures [24], MT1-MMP is known to mediate focal ECM degradation for the effective deposition of agrin in the presynaptic neurons, which in turn regulates AChR clustering in the postsynaptic muscle cells [43, 44]. Therefore, in agreement with our findings in the present study, MMP activity is dispensable for synaptic AChR clustering in muscle cells stimulated directly by agrin beads.

Since TrkB is primarily activated by mBDNF upon furin-mediated proteolytic conversion, we then examined the involvement of TrkB in AChR clustering by both overexpression (Fig. S4) and antisense MO-mediated knockdown (Fig. S5) approaches. In TrkB-EGFP-overexpressing muscle cells, we observed a dose-dependent inhibitory effect on the formation of topologically complex aneural AChR clusters (Fig. S4a, b), as well as the formation of synaptic AChR clusters induced by spinal neurons (Fig. S4c–e) and agrin beads (Fig. S4f–h). On the other hand, Western blot data showed that antisense TrkB MO effectively reduced the endogenous TrkB protein level in *Xenopus* myotomal tissue lysates (Fig. S5a, b). In TrkB MO muscle cells, synaptic AChR clustering induced by nerve innervation (Fig. S5c–e) and agrin bead stimulation (Fig. S5f–h) were both significantly inhibited. Taken together, our results indicated that furin-mediated proteolytic conversion of proBDNF to mBDNF is essential for the assembly of postsynaptic apparatus that involves mBDNF˗TrkB signaling.

### Muscle-generated BDNF is required for initial NMJ formation in vivo

**Fig. 8:**
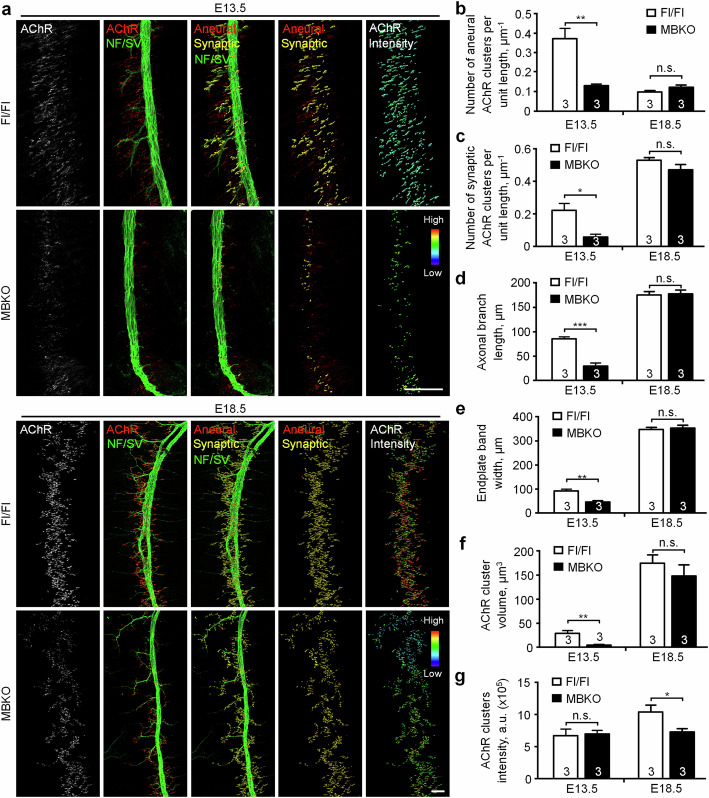
MBKO mice exhibit structural defects in the initial formation of NMJs in vivo. **a** Representative images showing aneural versus synaptic AChR clusters and their relative fluorescence intensity in whole-mount diaphragms between Fl/Fl control and MBKO mice at E13.5 and E18.5. The superimposed 3D reconstruction and surface rendering images (3rd column) were generated from z-stack images. Synaptic AChR clusters (yellow) were identified when the AChR and neurofilament (NF)/synaptophysin (SV) signals overlapped with each other, whereas other AChR signals (red) were classified as aneural AChR clusters. Pseudo-color images (5^th^ column) highlight the relative fluorescence intensity of AChR clusters. Quantification showing the number of aneural (**b**) versus synaptic (**c**) AChR clusters in diaphragm muscles per unit length of the main nerve trunk, the length of axonal branches (**d**), the width of end-plate bands (**e**), and the volume (**f**) and intensity (**g**) of all AChR clusters in diaphragm muscles per unit length of the main nerve trunk, between Fl/Fl and MBKO mouse embryos at E13.5 and E18.5. Scale bars represent 100 μm. Data are means ± SEM. The numbers indicated in the bar regions represent the total number of mouse embryos quantified from three independent experiments. *, **, *** represent *p* ≤ 0.05, 0.01, and 0.001, respectively (Student’s *t* test).

To explore the requirement of muscle-generated BDNF in the development of NMJs in vivo, we compared the structural features of NMJs in embryonic diaphragm muscles between MBKO and control floxed/floxed (Fl/Fl) mice at E13.5 and E18.5. During the initial NMJ formation in the diaphragm muscles at E13.5, MBKO mice exhibited a significantly lower density of both aneural and synaptic AChR clusters than Fl/Fl mice (Fig. 8a–c). Additionally, axonal defasciculation and arborization were affected in MBKO embryos, leading to a significant reduction in axonal branch length and endplate band width (Fig. 8d, e). Surprisingly, at E18.5, no significant differences in the number or volume of AChR clusters were detected between MBKO and Fl/Fl mice (Fig. 8b, c, f). The axonal branch length of the phrenic nerve and the endplate band width were also comparable between MBKO and Fl/Fl mice (Fig. 8d, e). We therefore hypothesize that muscle-generated BDNF is primarily involved in the initial formation of aneural and synaptic AChR clusters, but alternative sources of BDNF may compensate for its absence in MBKO mice to ensure the proper development of NMJs in vivo. Consistent with this hypothesis, recombinant BDNF treatment was able to rescue the inhibitory effects of muscle BDNF knockdown on agrin bead-induced AChR clustering in cultured muscle cells in dose- and time-dependent manners (Fig. S6). Nevertheless, although the number and volume of AChR clusters were relatively comparable between MBKO and Fl/Fl mice at E18.5, we still detected a significant reduction in the fluorescence intensity of AChR clusters in MBKO diaphragm muscles (Fig. 8g). These results were consistent with our in vitro findings showing the requirement of muscle BDNF for the initial formation of aneural AChR clusters, which in turn provide an immediate source of surface AChR molecules readily available for the redistribution to nascent synaptic sites during the assembly of nerve-induced postsynaptic apparatus.

## Discussion

In neuronal development, the neurotrophic hypothesis proposes that the spatiotemporally regulated release of trophic factors from target cells plays a crucial role in promoting the growth, survival, and differentiation of neurons [45]. Specifically, target-derived neurotrophins are known to regulate the guidance of neuronal growth cones, the formation and plasticity of synaptic connections, and the sculpturing of intricate nervous system circuitry [1, 46, 47]. Despite the initial well-characterized functions of BDNF in the brain, emerging evidence indicates that BDNF is also expressed in skeletal muscles and is crucial for muscle development, maintenance, and regeneration. In the early stages of muscle development, BDNF is found to be expressed in myoblasts, and its expression is repressed in differentiated myotubes [48]. In the maintenance and regeneration of adult skeletal muscles, BDNF regulates muscle adaptation and plasticity by contributing to the functional diversity and metabolic flexibility of skeletal muscles in response to exercise and other stimuli [49–53] and promotes the proliferation and differentiation of myosatellite cells during muscle repair and regeneration [54]. The regulation of BDNF signaling is therefore essential for proper muscle growth, formation, and function. Although some previous studies indicated that BDNF can modulate synaptic activity and function in NMJ development and disease [8, 12, 55], the cellular mechanisms underlying spatiotemporally controlled release of muscle-generated BDNF during the initial NMJ formation remain largely unknown.

**Fig. 9:**
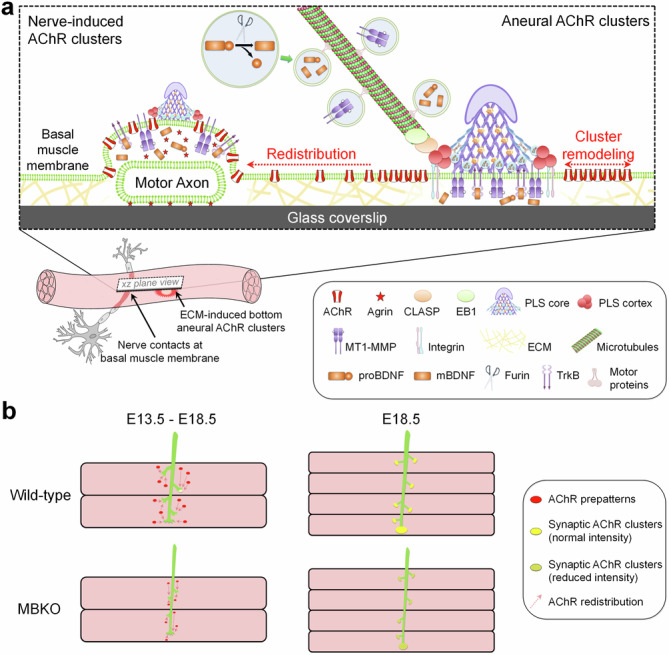
Site-directed vesicular trafficking and localized release of muscle-generated BDNF regulate AChR redistribution and clustering at developing NMJs in vitro and in vivo. **a** In nerve-muscle cocultures, we speculate that PLSs direct the vesicular trafficking of not only MT1-MMP, but also BDNF and its precursor proBDNF for localized release at AChR clusters. The intracellular endoprotease furin mediates the proteolytic conversion of proBDNF to mBDNF for activating TrkB receptors in muscles. Knockdown of muscle BDNF expression affects aneural AChR cluster formation; therefore, less AChR molecules are redistributed and recruited for the assembly of nerve-induced synaptic AChR clusters. **b** At developing NMJs in vivo, MBKO mouse embryos exhibit reduced AChR prepatterning in the diaphragm muscles and reduced axonal branching and arborization in the phrenic nerves at E13.5. The defects in AChR prepatterning reduce the number of AChR molecules being redistributed to the postsynaptic apparatus, as reflected by the reduced intensity of synaptic AChR clusters in MBKO mice, at E18.5.

In this study, we first demonstrated that BDNF is highly enriched at PLSs in association with the topologically complex postsynaptic apparatus in *Xenopus* muscle cells cultured on ECM-coated substratum (Fig. 1a), but no preferential localization is detected in simple plaque-shaped or scattered AChR clusters with no PLS localization in muscle cells cultured on uncoated or PDL substratum (Fig. S1b). This indicated the essential roles of ECM-mediated PLS formation in the subcellular localization of BDNF in muscle cells. PLSs are specialized actin-rich structures that play a crucial role in the site-directed trafficking of postsynaptic proteins to regulate the formation and topological remodeling of postsynaptic apparatus at NMJs [24, 27]. PLS is generally composed of an actin-rich core surrounded by a ring structure, consisting of cortex proteins to facilitate adhesion to the underlying substratum through integrins. At the nanoscale architecture of actin modularity, the core domain contains a central branched actin module encased by a linear actin module, while two actin modules (ventral filaments and dorsal interpodosomal filaments) are radiated from the core [56]. Our findings using Airyscan confocal super-resolution microscopy showed that endogenous BDNF is spatially enriched at the base of PLS core domain (Fig. 1d) and that its localization is positively correlated with the amount of newly polymerized F-actin and is inhibited by actin disrupting drug (Fig. 3). Additionally, high-potassium stimulation significantly reduces the spatially localized BDNF signals at the PLS core domain in aneural AChR clusters (Fig. 2). These data suggested that the spatial localization of BDNF is likely mediated by the ventral actin filament module of the PLS core, which may function to maintain BDNF vesicles within the reserve pool of secretable synaptogenic proteins for activity-dependent release (Fig. 9a).

Consistent with our previous studies on the vesicular trafficking of MT1-MMP [24], we here demonstrated that BDNF vesicles are transported to and captured at PLSs in ECM-induced aneural AChR clusters (Fig. 4a–c) and nerve-/agrin-induced synaptic AChR clusters (Fig. 5b, f). PLSs are known to mediate the intracellular trafficking of vesicles and surface targeting of newly synthesized membrane proteins via microtubule-based cargo delivery mechanisms [57, 58]. Using the pH-sensitive fluorescent protein SEP, a widely used fluorescence probe for imaging single-vesicle exocytosis [59], we further showed that the release of BDNF vesicles is spatially restricted occurring at aneural and synaptic AChR clusters (Figs. 4d, e, 5c, g). As revealed by live-cell time-lapse TIRF imaging, the transient bursts of BDNF-SEP signals that we observed at both aneural and synaptic AChR clusters indicate the localized release of vesicles containing diffusible BDNF proteins into the immediate extracellular environment. These results demonstrated the essential roles of PLSs in directing not only the vesicular trafficking and surface insertion of transmembrane proteins, such as MT1-MMP and AChR [23, 24, 39], but also the vesicular trafficking and release of the secretable protein BDNF, at the postsynaptic apparatus (Fig. 9a). Although PLSs are normally found in topologically complex postsynaptic apparatus at postnatal NMJs, our findings showed that site-directed trafficking and localized release of BDNF are also detected at the periphery of AChR clusters (Fig. 4c–e), where only PLS cortex markers are concentrated (Fig. 1c), in cultured *Xenopus* muscle cells. Therefore, we speculate that the presence of PLS cortex proteins may be sufficient for directing the vesicular trafficking and localized release of BDNF at AChR clusters during the embryonic development of NMJs in vivo, in which the distinctive structure of entire PLSs may not be clearly defined.

The proteolytic conversion of proBDNF to mBDNF is an important step in the regulation of BDNF signaling and function, and this process is tightly regulated by different intracellular and extracellular proteases. In human skeletal muscles, proBDNF is known to be much more abundant than mBDNF [60]. Consistent with this, our Western blot data also showed that the expression level of mBDNF is relatively lower than that of proBDNF and is modestly reduced by BDNF MO in *Xenopus* myotomal tissue lysates (Fig. 6a). Our immunostaining data, however, showed a significant reduction of both proBDNF and BDNF signals at AChR clusters in BDNF MO muscle cells (Fig. 6c, f). More importantly, the formation of ECM-induced aneural AChR clusters (Fig. 6d) and nerve-/agrin-induced AChR clusters (Fig. 7a–e) were significantly inhibited by MO-mediated BDNF knockdown in muscles. Since no noticeable changes in the expression of muscle-specific kinase (MuSK), a receptor tyrosine kinase that is crucial for nerve-independent AChR prepattern formation [61, 62], were detected between wild-type and BDNF MO muscles (Fig. S7), the inhibitory effects of BDNF knockdown on AChR clustering are unlikely caused by the general deficits in muscle development or postsynaptic protein expression. These findings indicated that the dysregulation of subcellular localization of BDNF and the imbalanced actions between proBDNF and mBDNF signaling drastically affect the proper assembly of postsynaptic apparatus.

A previous study showed that MMP activity mediates the proteolytic conversion of proBDNF to mBDNF, which differentially regulates synapse elimination versus stabilization at polyinnervated NMJs in postnatal mice [21]. Interestingly, our findings showed that inhibiting furin-mediated intracellular proteolytic conversion, but not inhibiting MMP-mediated extracellular proteolytic conversion, significantly decreased BDNF signals and increased proBDNF signals (Fig. 6g–i). In addition, furin inhibition also suppressed the formation of synaptic AChR clusters induced by spinal neurons (Fig. 7i, j) or agrin beads (Fig. 7f–h). These inhibitory effects on nerve-/agrin-induced synaptic AChR clustering were comparable in BDNF MO muscle cells with reduced expression of both proBDNF and mBDNF (Figs. 6a, b and 7a–e). Upon proteolytic conversion, mBDNF binds to and activates TrkB for initiating intracellular signaling cascades that regulate synaptic structure and functions. In agreement with previous studies on the disruption of TrkB-mediated signaling in cultured myotubes and adult NMJs [63], our findings also showed that either TrkB-EGFP overexpression (Fig. S4) or antisense MO-mediated TrkB knockdown (Fig. S5) significantly inhibits nerve- or agrin-induced AChR clustering, mirroring the effects of BDNF knockdown or furin inhibition (Fig. 7). Taken together, we hypothesize that the proteolytic conversion of proBDNF to mBDNF is precisely controlled to modulate their distinct autocrine functions at the vertebrate NMJs in different developmental stages: furin-mediated intracellular conversion regulates the initial formation of postsynaptic apparatus in embryonic development (as reported in this study); and MMP-mediated extracellular conversion regulates synaptic competition and elimination in the polyinnervated postsynaptic apparatus in postnatal development [21, 22].

In this study, the physiological involvement of muscle BDNF in NMJ development in vivo is further validated by examining the aneural and synaptic AChR clusters in the diaphragm muscles of MBKO mouse embryos. Prior to innervation by motor neurons, aneural AChR clusters, termed AChR prepatterns, can be detected in the central region of muscle fibers at E13.5 [61, 62]. In zebrafish, these prepatterns may guide motor axons for making synapses at the early phase of NMJ formation [64]. While prepatterning has a transitory function for positioning the nerve on the synapse [65], it is also believed that a significant amount of AChR molecules from the dispersing prepatterns are subsequently recruited to the nascent postsynaptic sites, partly contributing to the high receptor density of 10,000/µm^2^ in the postsynaptic apparatus [66]. In this study, we found that the number of both aneural AChR prepatterns and synaptic AChR clusters in MBKO diaphragm muscles were significantly lower than those in Fl/Fl mice at E13.5 (Fig. 8a–c), indicating that muscle-generated BDNF is required for the initial formation of aneural AChR prepatterns, which may reduce the availability of AChR molecules for being recruited to the nascent synaptic sites during early NMJ development in vivo (Fig. 9b). It is of note that the neurotransmitter acetylcholine is known to disperse AChR prepatterns that are not stabilized by nerve innervation, raising the possibility of retrograde functions of muscle BDNF in regulating acetylcholine release from the motor neurons. However, given the well-characterized effects of BDNF on the potentiation of neurotransmitter release from presynaptic nerve terminals at the NMJs and many other synapses [67–69], the defects in AChR prepatterning observed in early MBKO mouse embryos are unlikely caused by increased acetylcholine release from the motor neurons, leading to exuberant elimination of AChR prepatterns. Additionally, compared to the age-matched Fl/Fl mice, MBKO mice also exhibited a markedly reduced axonal length in the phrenic nerve as reflected by staining with a mixture of neurofilament and synaptophysin antibodies, leading to a narrower endplate band width (Fig. 8d, e). These observations are consistent with the well-known functions of BDNF in promoting axonal growth and branching [13, 70–72].

At E18.5, while we detected reduced AChR cluster intensity in MBKO mice (Fig. 8g), intriguingly no significant differences were detected in the axonal length of presynaptic neurons and the number of aneural and synaptic AChR clusters in postsynaptic muscles (Fig. 8b–f). In agreement with our findings, a previous study that examined the localization of different postsynaptic proteins and the apposition of presynaptic and postsynaptic elements at adult NMJs reveals no significant reduction on muscle BDNF deletion [52]. While we do not rule out the possibility that muscle BDNF is dispensable for maintaining NMJ integrity in adult animals as suggested in that study, we speculate that alternative sources of BDNF may compensate for the inhibitory effects of muscle BDNF deficiency on synaptic development in both presynaptic neurons and postsynaptic muscles, considering that BDNF is also expressed in motor neurons, perisynaptic Schwann cells, and endothelial cells [73–75]. In fact, recombinant BDNF treatment is capable of restoring agrin bead-induced AChR clustering in BDNF MO muscles in dose- and time-dependent manners (Fig. S6), further supporting our hypothesis that diffusible BDNF proteins secreted from the alternative non-muscle sources may rescue the structural defects of NMJs in MBKO mice at late embryonic and postnatal stages.

On the other hand, our recent works also reported that the ability of fasting-induced fuel source switching and the maintenance of mitochondrial activity and function are greatly impaired in adult MBKO mice [50, 51]. Therefore, in addition to the involvement in initial NMJ formation at early embryonic stages between E13.5 and E18.5 (Fig. 8), muscle-generated BDNF also functions as a myokine to optimize the metabolic properties of adult skeletal muscles during nutrient stress via maintaining mitochondrial quality. In summary, this present study reported the cell biological mechanisms underlying the vesicular trafficking, proteolytic processing, and spatially restricted release of BDNF in skeletal muscles and revealed a previously unappreciated role of muscle-generated BDNF in regulating the initial formation of aneural AChR clusters for the subsequent assembly of postsynaptic apparatus during the early development of NMJs in vitro and in vivo.

## Materials and methods

### Preparation of *Xenopus* primary muscle and nerve-muscle cultures

Adult *Xenopus laevis* animals were purchased from Xenopus 1. *Xenopus* eggs were fertilized in vitro, and the embryos were raised in 10% Holtfreter’s solution (60 mM NaCl, 0.6 mM KCl, 0.9 mM CaCl_2_, and 0.2 mM NaHCO_3_; pH 7.4) at 22 °C [23]. 460 pg DNA construct encoding BDNF-SEP (Addgene, 83955; RRID: Addgene_83955), 46-92 pg DNA construct encoding GFP-cortactin (Addgene, 26722; RRID: Addgene_26722), or 3.5–7.0 ng mRNA construct encoding BDNF-mCherry was injected into one-cell stage *Xenopus* embryos using Nanoject II (Drummond Scientific). mCherry- or SEP-expressing embryos were screened and selected for primary culture preparation. The dorsal parts of the embryos at the Nieuwkoop and Faber stages 19–22 were dissected, and then subjected to enzymatic digestion using collagenase (Sigma-Aldrich, C98191G) as previously described [76]. The myotomal tissues and neural tubes were collected, followed by dissociation with calcium-magnesium-free solution. The dissociated cells were plated on glass coverslips or glass-bottom plastic dishes coated with a mixture of cell attachment substrate, entactin-collagen IV-laminin (Millipore, 08-100). Cultured cells were maintained in culture medium containing 87% Steinberg’s solution (v/v, 60 mM NaCl, 0.67 mM KCl, 0.35 mM Ca(NO_3_)_2_, 0.83 mM MgSO_4_, and 10 mM HEPES), 10% Leibovitz’s L-15 medium (v/v), 1% fetal bovine serum (v/v), 1% penicillin-streptomycin (v/v), and 1% gentamicin sulfate (v/v). Muscle cultures were maintained at 22 °C for at least 2 days to facilitate cell attachment and aneural AChR cluster formation before pharmacological treatment, if any. To induce postsynaptic differentiation, polystyrene latex beads coated with recombinant agrin (R&D Systems, 550-AG-100/CF) or dissociated spinal neurons were added to 2 day-old muscle cultures as previously described [13, 23] and then maintained for the specified time before performing live-cell imaging. All the experiments involving *Xenopus* frogs and embryos were carried out in accordance with the protocols approved by the Research Ethics Committee at Hong Kong Baptist University.

#### Morpholino-mediated knockdown of endogenous proteins

The knockdown expression of endogenous proteins in *Xenopus* embryos was achieved by microinjecting custom-designed antisense MO (Gene Tools), which binds to the target mRNA sequence to block its translation. Two different antisense BDNF MO sequences (5ʹ-ATG GTC ATC ACT CTT CTC ACC TGA-3ʹ and 5ʹ-CTC ACC TGA TGG AAC TTA TTT TAG C-3ʹ) and an antisense TrkB MO sequence (5ʹ-CCA GAG GCG CAT GGT GGA TCT CCG G-3ʹ) were used in this study. To identify the presence of MO in the cultured cells, the cell lineage tracer Alexa Fluor 488-conjugated dextran (Thermo Fisher Scientific, D22910) was co-injected into the embryos. The knockdown efficiency of endogenous protein expression in myotomal tissues and cultured muscle cells from MO-injected embryos was validated by Western blot and immunostaining experiments, respectively.

#### Pharmacological treatment

To study the release mechanisms of spatially localized BDNF at aneural AChR clusters, muscle cultures were treated with 50 mM high potassium solution for 5 min with or without 1 μM TTX (Hello Bio, HB1035) or 25 μM BAPTA-AM (ApexBio, B4758) for 30 min. To study the colocalization between BDNF and newly synthesized F-actin, muscle cultures were pre-treated with 2.5 μM jasplakinolide (Santa Cruz Biotechnology, sc-202191) for 3 h to mask all pre-existing F-actin structures, followed by extensive washing with the culture medium. After overnight recovery in drug-free culture medium, newly synthesized F-actin was then probed with fluorescent phalloidin [33]. To study the effects of actin polymerization inhibition on BDNF localization, muscle cultures were treated with 10 μM Ltn A (Abcam, ab144290) for 4 h. To study the involvement of MMP and furin in the proteolytic conversion of proBDNF to mBDNF, muscle cultures were treated with 5 μM BB-94 (ApexBio, A2577) or 10 μM furin inhibitor (EMD Millipore, 344930) for 4 h. For the experiments involving agrin bead or nerve stimulation, treatment with BB-94 or furin inhibitor was started approximately 1 h before the addition of agrin beads or spinal neurons to the cultured muscle cells.

#### Live-cell staining, cell fixation, and immunostaining

To label the AChR clusters, muscle cultures or nerve-muscle co-cultures were incubated with 0.1 μM tetramethylrhodamine- or Alexa Fluor 647-conjugated α-bungarotoxin (Thermo Fisher Scientific, T1175 or B35450) for 45 min, followed by extensive washing with culture medium. The samples were then immediately used for live-cell imaging or cell fixation for subsequent immunostaining experiments.

Cultured muscle cells from wild-type embryos were fixed with 4% paraformaldehyde in PBS for 15 min, and those from microinjected embryos with fluorescence protein tags were fixed with 1% paraformaldehyde in PBS for 20 min. The fixed cells were then permeabilized with 0.1% Triton X-100 for 10 min, followed by extensive washing with PBS at least 3 times. After blocking with 2% bovine serum albumin (Sigma-Aldrich, A9418) at 4 °C overnight, the samples were incubated with primary antibodies at room temperature for 2 h, followed by staining with fluorophore-conjugated secondary antibodies (1:400; Thermo Fisher Scientific) for 45 min, and/or Alexa Fluor 633-conjugated phalloidin (1:1000; Thermo Fisher Scientific, A22284) for 45 min. The primary antibodies used in this study include BDNF antibody (1:1000; Alomone Labs, ANT-010; RRID: AB_2039756), proBDNF antibody (1:1500; Alomone Labs, ANT-006; RRID: AB_2039758), TrkB antibody (1:200; Santa Cruz Biotechnology, sc-119; RRID: AB_632559), phospho-TrkB (Tyr705) antibody (1:200; Thermo Fisher Scientific, PA5-38077; RRID: AB_2554680), cortactin antibody (1:1200; EMD Millipore, 05-180; RRID: AB_309647), and vinculin antibody (1:100; DSHB, VN 3-24; RRID: AB_2214518). Coverslips were then mounted on glass slides with the anti-bleaching reagent fluoromount-G (Thermo Fisher Scientific, 00-4958-02) for later observation.

#### Western blot analysis

Myotomal tissues from *Xenopus* embryos at Nieuwkoop and Faber stage 19–22 were homogenized in radioimmunoprecipitation assay buffer supplemented with protease inhibitor cocktail and EDTA, and subsequently incubated on ice for 5 min. After high-speed centrifugation at 15,000 × *g*, the supernatant fraction was collected for protein concentration determination using a BCA protein assay kit (Thermo Fisher Scientific, 23227). 30 μg protein lysates were separated by SDS–polyacrylamide gel electrophoresis (SDS–PAGE) using the mini-PROTEAN Tetra (Bio-Rad Laboratories), and then transferred to polyvinylidene difluoride (PVDF) membranes using the Trans-Blot Turbo Transfer System (Bio-Rad Laboratories). After blocking with 3% BSA in Tris-buffered saline with tween 20 (TBST), the blots were probed with the following primary antibodies: anti-BDNF (1:500, abcam, ab108319; RRID: AB_10862052), anti-TrkB (C-13) (1:500, Santa Cruz Biotechnology, sc-119; RRID: AB_632559), MuSK (C-19) (1:1000, Santa Cruz Biotechnology, sc-6009; RRID: AB_2266741), anti-α-tubulin (1:10000; Sigma-Aldrich, T6074; RRID: AB_477582), or anti-β-tubulin (1:1000, DSHB, E7; RRID: AB_528499) at 4 °C overnight. After extensive washing with TBST, the blots were probed with horseradish peroxidase-conjugated secondary antibodies. After incubating the blots with ECL Western blotting substrate (Thermo Fisher Scientific, 32106), signals were detected by using a ChemiDoc XRS+ system (Bio-Rad Laboratories).

### Preparation of whole-mount diaphragm muscles from mouse embryos

MBKO mice were generated by crossing BDNF *Flox/Flox* mice (Jackson Laboratory, 004339; RRID: IMSR_JAX:004339) with human α-skeletal actin-promoter-driven Cre transgenic mice (HSA-Cre; Jackson Laboratory, 006149; RRID: IMSR_JAX:006149). PCR was performed for genotyping the genomic DNA extracted from the tail using specific primers suggested by the Jackson Laboratory. For experiments involving whole-mount diaphragm muscle tissues, the entire diaphragm was dissected from mouse embryos and then fixed in 2% paraformaldehyde in PBS at room temperature for 1 h, followed by incubating with 0.1 M glycine in PBS for 30 min. The fixed tissues were labeled with 0.1 μM tetramethylrhodamine α-bungarotoxin for 45 min. After extensive washing with PBS, the tissues were labeled with a mixture of neurofilament NF200 (1:2000; Sigma-Aldrich, N4142; RRID: AB_10862052) and synaptophysin (1:2000; Abcam, ab32127; RRID: AB_2286949) antibodies at 4 °C for 2 days, followed by incubation overnight with Alexa Fluor 488-conjugated secondary antibodies. The samples were flat-mounted with Dapi-Fluoromount-G (Electron Microscopy Sciences, 17984-24) onto glass slides. All the experiments involving mouse embryos were carried out in accordance with the protocols approved by the Committee on the Use of Live Animals in Teaching and Research at The University of Hong Kong or the Research Ethics Committee at Hong Kong Baptist University.

#### Fluorescence microscopy

Wide-field fluorescence imaging was performed on Olympus IX83 inverted microscope using an oil-immersion Plan Apo 60× N.A. 1.42 objective lens (Evident) or Nikon Eclipse Ti2-E inverted microscope using an oil-immersion CFI Plan Apochromat Lambda D 60× N.A. 1.42 objective lens (Nikon Instruments). Digital still images were captured by either ORCA Flash4.0 LT+ (Hamamatsu) or Zyla 4.2 (Andor) sCMOS cameras through MicroManager software (Open Imaging) [77].

For the FRAP experiments, TIRF mode was used with an Axio TIRF unit fitted on Nikon Eclipse Ti2-E inverted microscope using an oil-immersion CFI Apo TIRF 100× N.A. 1.49 objective lens (Nikon Instruments). In BDNF-mCherry-/GFP-cortactin-expressing muscle cells, the regions of interest (ROIs) identifying aneural AChR clusters or agrin bead-/nerve-induced AChR clusters were selected for photobleaching. Images were captured by an Evolve 512 EMCCD camera (Photometrics) through Metamorph software (Molecular Devices).

To visualize the immunostaining signals of BDNF, proBDNF, and cortactin at aneural AChR clusters, multiple z-stack images were captured on LSM 880 with Airyscan confocal microscope using an oil-immersion 63× N.A. 1.40 objective lens (Carl Zeiss). For mouse diaphragm muscle samples, tile-scanned z-stack images were captured on LSM 800 confocal microscope using a 20× N.A. 0.80 objective lens (Carl Zeiss). Images were captured by 32-channel GaAsp photomultiplier modules through ZEN 2.3 software (Carl Zeiss).

All acquisition settings (i.e. laser intensity and gain) were kept the same for different experimental groups in the same experiment. Confocal z-stack images were subjected to Airyscan processing if applicable. All the acquired images were processed and analyzed using ImageJ (National Institute of Health) or Imaris (Oxford Instruments) software.

#### Data analysis

The percentage of perforated AChR clusters with respective protein marker localization was quantified by scoring the presence of spatially enriched fluorescence signals at aneural AChR clusters in cultured muscle cells. For quantifying the fluorescence intensities of intracellular PLS-localized protein markers (BDNF, proBDNF, F-actin, and cortactin), ROIs were created at the perforated regions of aneural AChR clusters. They were then applied to the fluorescence images of respective protein markers for measurement. For quantifying the fluorescence intensities of TrkB and pTrkB that are highly colocalized with AChR clusters, ROIs were created at the entire AChR clusters and then applied to TrkB/pTrkB fluorescence images for measurement. The intensity values were then normalized to the background intensity measured using the same ROIs.

The percentage of bead-/nerve-muscle contacts with AChR clustering was quantified by scoring the presence of AChR clusters at the physical contacts between the muscle cells and agrin beads or spinal neurons that were identified in the phase contrast images. For calculating the normalized intensity of aneural AChR clusters, the measured intensity in each experimental groups was normalized against the average AChR intensity in the control condition of the same experiment. For calculating the normalized intensity of agrin bead-induced AChR clusters, AChR intensity at the bead-muscle contacts was first measured in each experimental groups and then normalized against the values in the 4-h control condition in the same experiment. For calculating the normalized intensity of nerve-induced AChR clusters, AChR integrated intensity at the nerve-muscle contacts was first measured and then divided by the length of the contacts. After that, they were normalized against the values in the control group.

For quantifying the density of aneural and synaptic AChR clusters in embryonic mouse diaphragm muscles, the total number of AChR clusters was first counted from z-stack images using Imaris. Synaptic AChR clusters were determined by the overlap of signals between AChR clusters and neurofilaments. The number of aneural AChR clusters was calculated by subtracting the number of synaptic clusters from the total number of AChR clusters. The calculated numbers were then normalized to the length of the main nerve trunk. The endplate band width was quantified by measuring the average distance between two farthest AChR clusters in the diaphragm muscles along the main nerve trunk. The length of the nerve branches was quantified by measuring the average axonal length from the main nerve trunk. The volume and intensity of AChR clusters were measured using the surface rendered images generated in Imaris. The values of AChR volume were normalized to the length of the main nerve trunk, while the values of AChR intensity were normalized to the total AChR volume.

In all the figures, the mean and SEM values are shown, unless otherwise specified. The numbers of biological replicates and the statistical tests applied are specified in the figure legends. Data analyses were performed using Prism 9 (GraphPad Software).

## Supplementary information

Supplementary Figures S1 - S7

Original data

## Data Availability

All data needed to evaluate the conclusions in the paper are present in the paper and/or the Supplementary Materials.
