# Supplementary material for: Localized release of muscle-generated BDNF regulates the initial formation of postsynaptic apparatus at neuromuscular synapses
Source: Cell Death Differ. 2024 Nov 7;32(3):546–60. doi: 10.1038/s41418-024-01404-4 (PMC11893767; doi:10.1038/s41418-024-01404-4)
Supplement: Supplementary file 1 — Supplementary Figures S1 - S7 [file 41418_2024_1404_MOESM1_ESM.pdf]

Localized Release of Muscle-Generated BDNF Regulates the Initial Formation of Postsynaptic Apparatus at Neuromuscular Synapses

Supplementary Figures

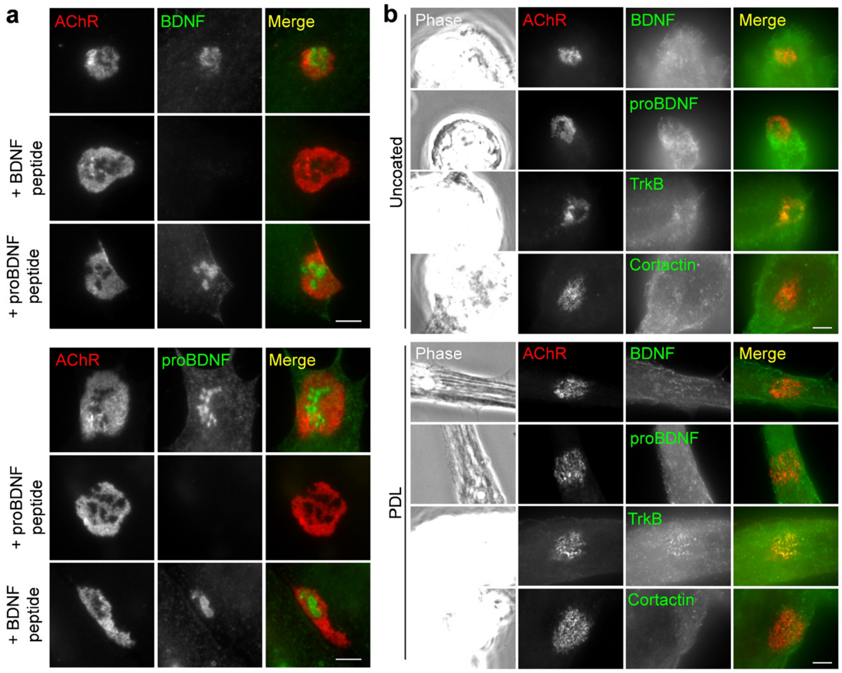

Figure S1. The specificity of BDNF and proBDNF immunostaining signals validates their spatial localization at ECM-induced aneural AChR clusters.

(**a**) Representative images showing the spatially localized BDNF and proBDNF signals at aneural AChR clusters can be completely abolished by pre-incubating the primary antibodies with their respective immunogenic peptides against BDNF and proBDNF, respectively, but not vice versa.

(**b**) Representative images showing no preferential localization of BDNF, proBDNF, TrkB, and cortactin at AChR clusters in muscle cells cultured on uncoated (top panels) or PDL-coated (bottom panels) substrates. Phase contrast images show that most cells exhibit myoball morphology in the uncoated condition.

Scale bars represent 5 µm.

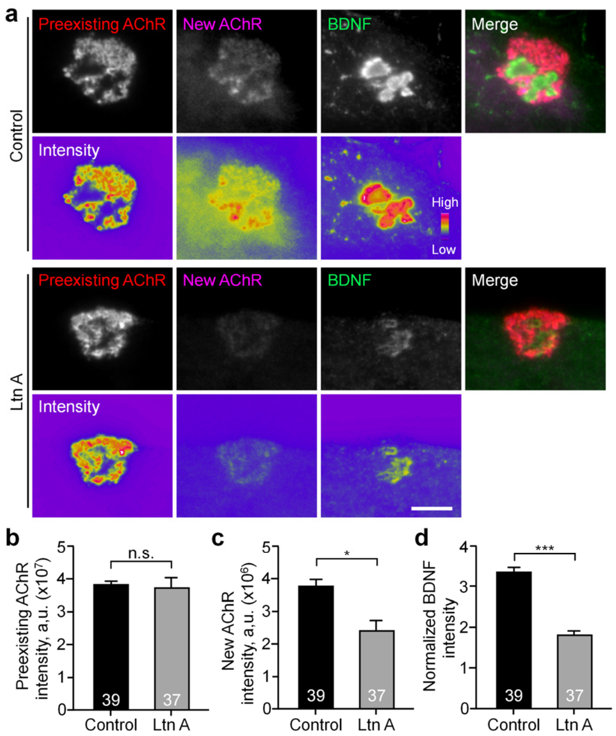

Figure S2. Pharmacological inhibition of actin polymerization affects spatial localization of BDNF and surface insertion of new AChR molecules.

(**a**) Representative images showing that the newly inserted AChR molecules, together with the spatially localized BDNF signals, are significantly reduced in muscle cells treated with Ltn A for 4-h. In contrast, the stability of preexisting AChR clusters was largely unaffected during that period. Preexisting and newly inserted AChR molecules were differentially labeled with α-bungarotoxin conjugated with different fluorophores in accordance with our previously published procedures [1].

(**b – d**) Quantification showing the effects of Ltn A treatment on the intensities of preexisting AChR clusters (b), newly inserted AChR molecules (c), and BDNF (d) at the perforated aneural AChR clusters.

Scale bar represents 5 μm. 8-bit pseudo-color images highlight the relative fluorescence intensity. Data are mean ± SEM. The numbers indicated in the bar regions represent the total numbers of muscle cells quantified from 3 independent experiments. *, *** represent $p \leq 0.05$, and 0.001, respectively (Student's t-test). n.s.: non-significant.

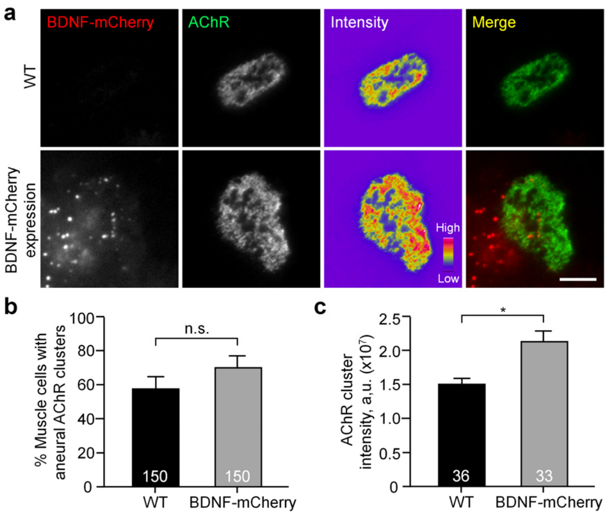

Figure S3. BDNF-mCherry expression at low levels modestly promotes AChR clustering.

(**a**) Representative images and quantification (**b, c**) showing that the modest effects of low BDNF-mCherry expression on AChR clustering in cultured muscle cells.

Scale bar represents 5 μm. 8-bit pseudo-color images highlight the relative fluorescence intensity. Data are mean ± SEM. The numbers indicated in the bar regions represent the total numbers of muscle cells quantified from 3 independent experiments. * represents $p \leq 0.05$ (Student's t-test). n.s.: non-significant.

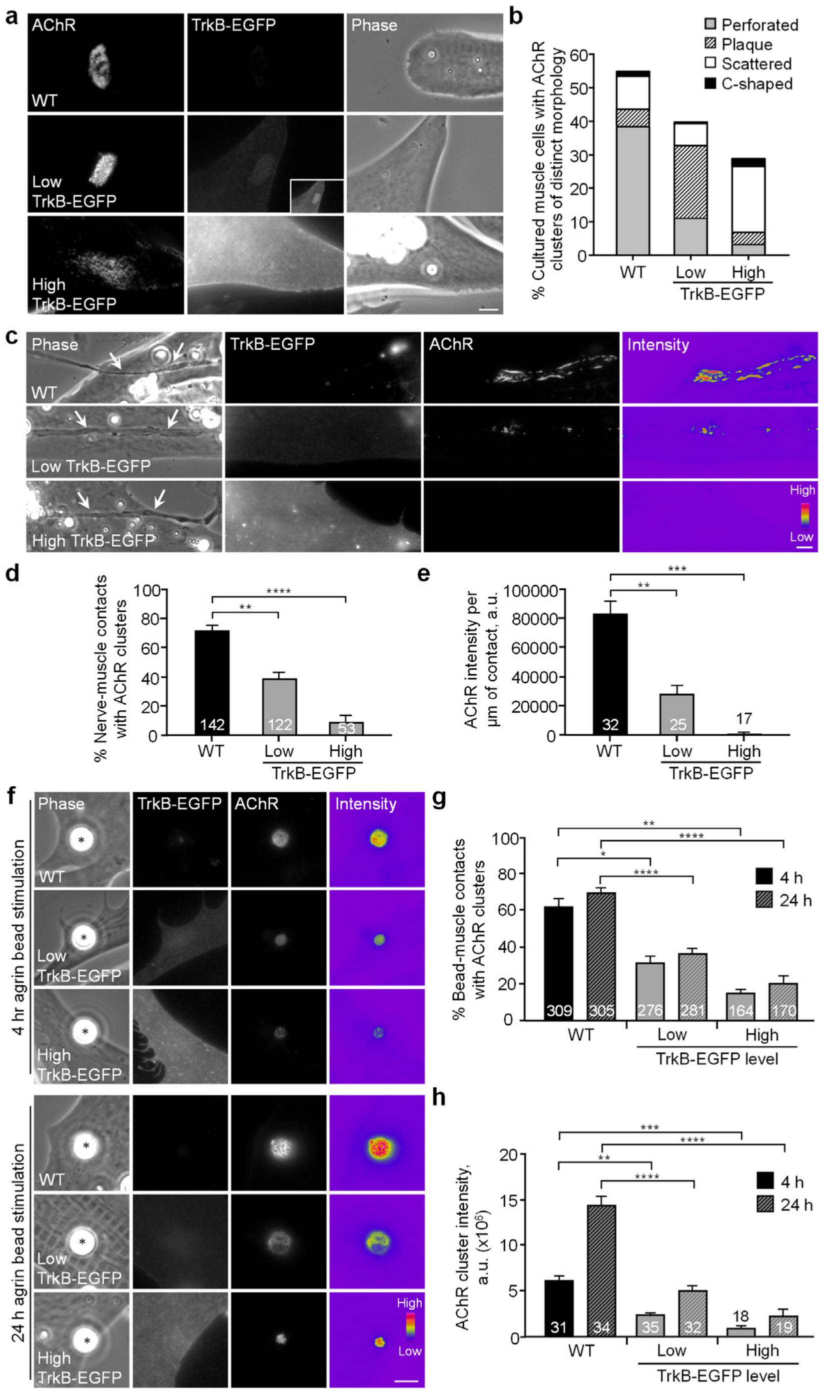

Figure S4. TrkB overexpression affects the formation of topologically complex aneural AChR clusters and nerve-/agrin-induced AChR clusters.

(**a, b**) Representation images (a) and quantification (b) showing the dose-dependent effects of TrkB-EGFP overexpression on the formation of topologically complex aneural AChR clusters in cultured muscle cells. Inset: the contrast-enhanced images of TrkB-EGFP showing the co-localization of TrkB-EGFP and AChR clusters.

(**c – e**) Representative images (c) and quantification showing the effects of TrkB-EGFP overexpression on nerve-induced AChR clustering (d) and their integrated fluorescence intensity per unit length of nerve-muscle contacts (e). Arrows indicate nerve-muscle contacts.

(**f – h**) Representative images (f) and quantification showing the effects of TrkB-EGFP overexpression on the formation of agrin bead-induced AChR clusters (g) and their integrated fluorescence intensity (h) after 4- or 24-hour stimulation.

Scale bars represent 5 µm. Asterisks indicate bead-muscle contacts. 8-bit pseudo-color images highlight the relative fluorescence intensity. Data are means ± SEM. The numbers indicated in the bar regions represent the total numbers of nerve-muscle contacts (d and e) and bead-muscle contacts (g and h) measured from three independent experiments. *, **, ***, **** represent $p \leq 0.05$, 0.01, 0.001, and 0.0001, respectively (one-way ANOVA with Tukey's multiple comparisons test).

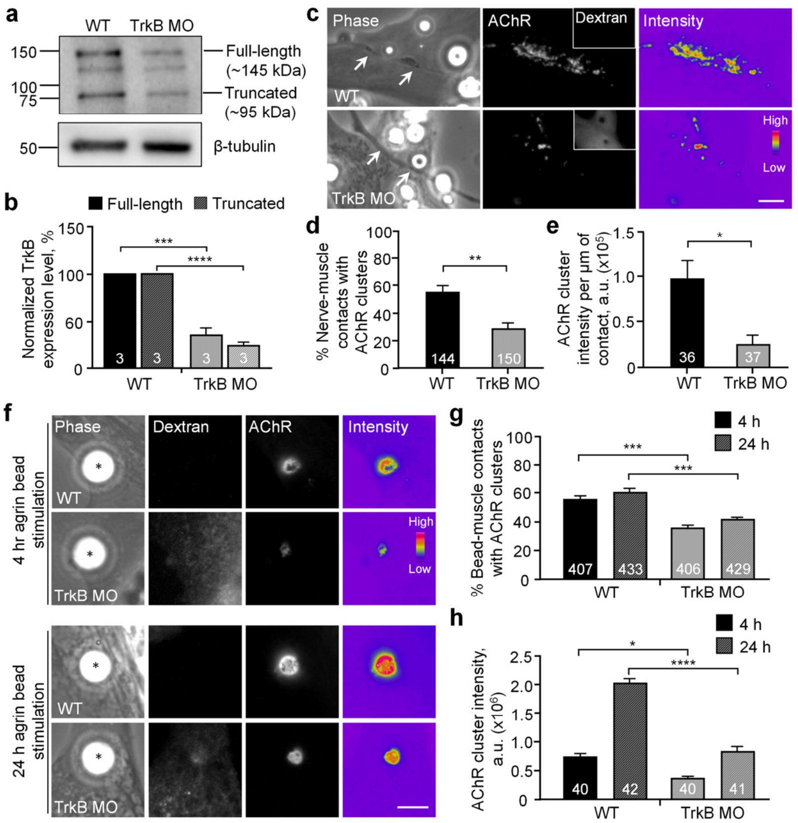

Figure S5. TrkB knockdown affects synaptic AChR cluster formation induced by spinal neurons or agrin beads.

(**a, b**) Western blot analysis (a) and quantification (b) showing the effective knockdown of both full-length and truncated forms of TrkB expression by antisense TrkB MO. WT: wild-type. Anti-β-tubulin was used as a loading control.

(**c – e**) Representative images (c) and quantification showing the effects of TrkB MO on nerve-induced AChR clustering (d) and their integrated fluorescence intensity per unit length of nerve-muscle contacts (e). Arrows indicate nerve-muscle contacts.

(**f – h**) Representative images (f) and quantification showing the effects of TrkB MO on the formation of agrin bead-induced AChR clusters (g) and their integrated fluorescence intensity (h) after 4- or 24-hour stimulation.

Scale bars represent 5 μm. Asterisks indicate bead-muscle contacts. Fluorescent dextran signals indicate the presence of TrkB MO in muscle cells. 8-bit pseudo-color images highlight the relative fluorescence intensity. Data are means ± SEM. The numbers indicated in the bar regions represent total numbers of blots (b), nerve-muscle contacts (d and e) and bead-muscle contacts (g and h) measured from three independent experiments. *, **, ***, **** represent $p \le 0.05$, 0.01, 0.001, and 0.0001, respectively (Student's t-test (b, d, and e) or one-way ANOVA with Tukey's multiple comparisons test (g and h)).

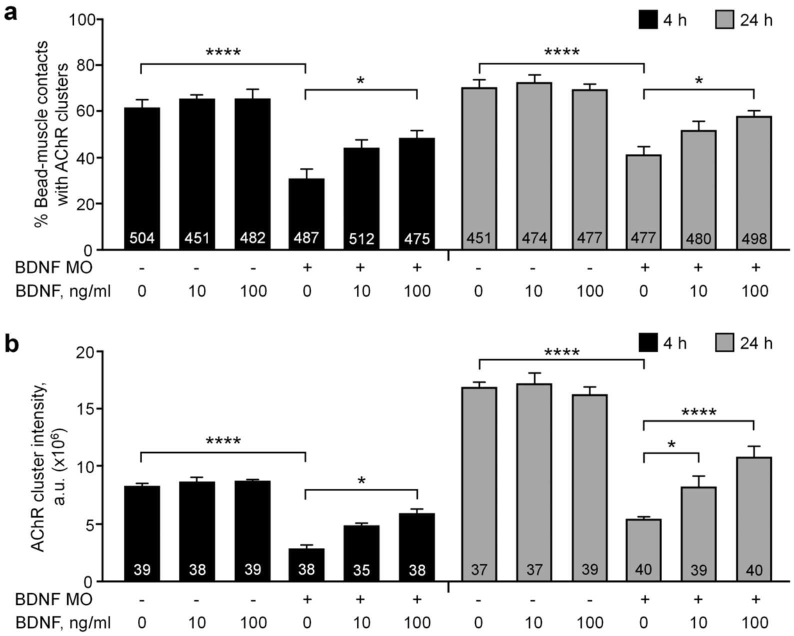

Figure S6. Recombinant BDNF treatment restores agrin-induced AChR clustering in BDNF MO muscle cells. Quantification showing the dose- and time-dependent effects of recombinant BDNF treatment on the formation of agrin bead-induced AChR clusters (a) and their normalized fluorescence intensity of AChR signals at bead-muscle contacts (b) in BDNF MO muscle cells. Data are means ± SEM. The numbers indicated in the bar regions represent the total numbers of bead-muscle contacts measured from three independent experiments. *, **** represent $p \leq 0.05$, and 0.0001, respectively (one-way ANOVA with Tukey's multiple comparisons test).

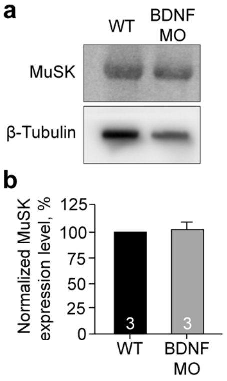

Figure S7. BDNF knockdown does not affect the expression of MuSK. Western blot analysis (a) and quantification (b) showing the comparable amount of MuSK protein expression between wild-type (WT) and BDNF MO muscle tissues. Anti-β-tubulin was used as a loading control.

Data are means ± SEM. The numbers indicated in the bar regions represent total numbers of blots measured from three independent experiments.

Reference

1. Lee CW, Han J, Bamburg JR, Han L, Lynn R, Zheng JQ. Regulation of acetylcholine receptor clustering by ADF/cofilin-directed vesicular trafficking. *Nat Neurosci* 2009, **12**(7)**:** 848-856.
