# Supplementary material for: Localized release of muscle-generated BDNF regulates the initial formation of postsynaptic apparatus at neuromuscular synapses
Source: Cell Death Differ. 2024 Nov 7;32(3):546–60. doi: 10.1038/s41418-024-01404-4 (PMC11893767; doi:10.1038/s41418-024-01404-4)

Full-length Western blot in Fig. 6a

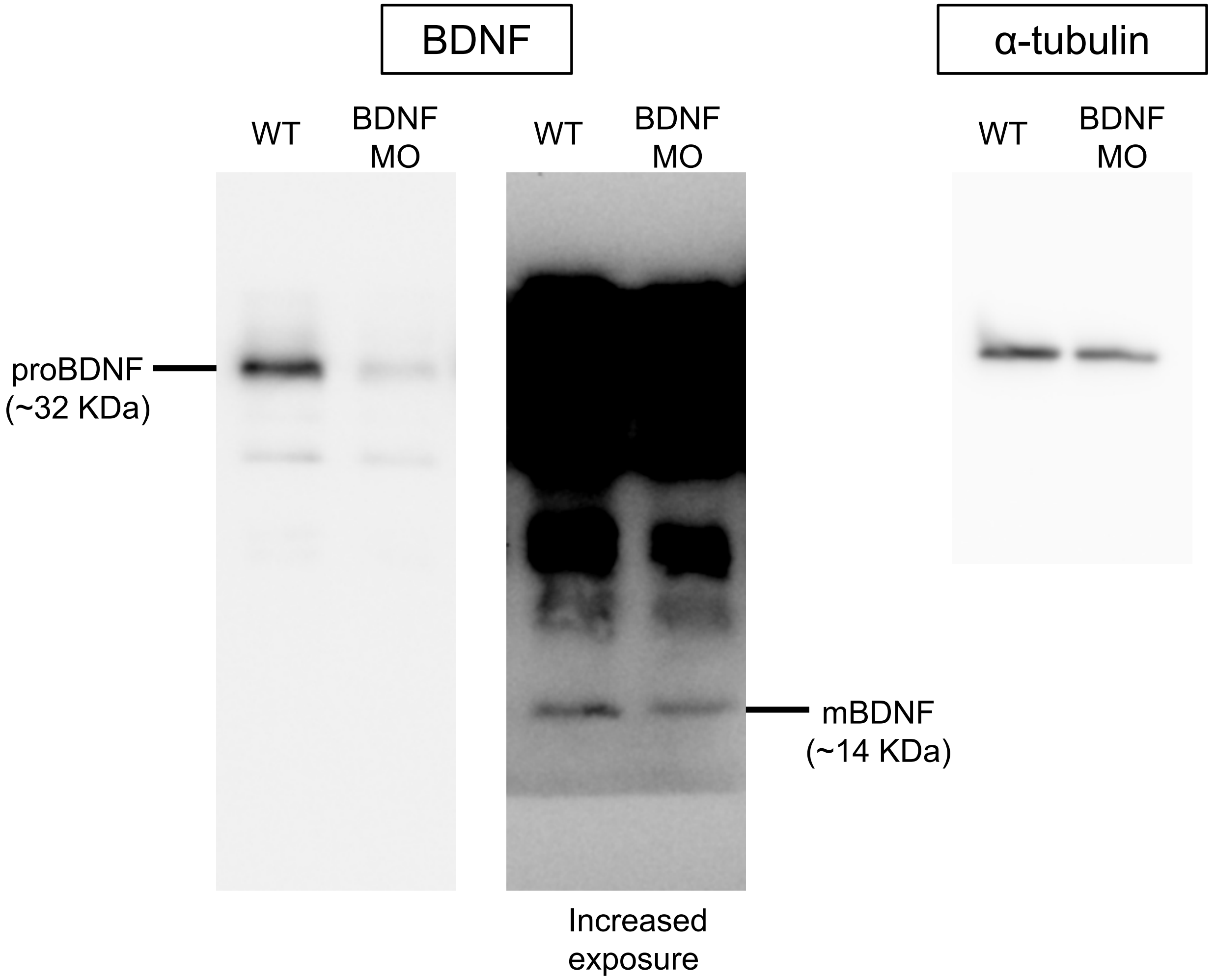

Full-length Western blot in Fig. S5a

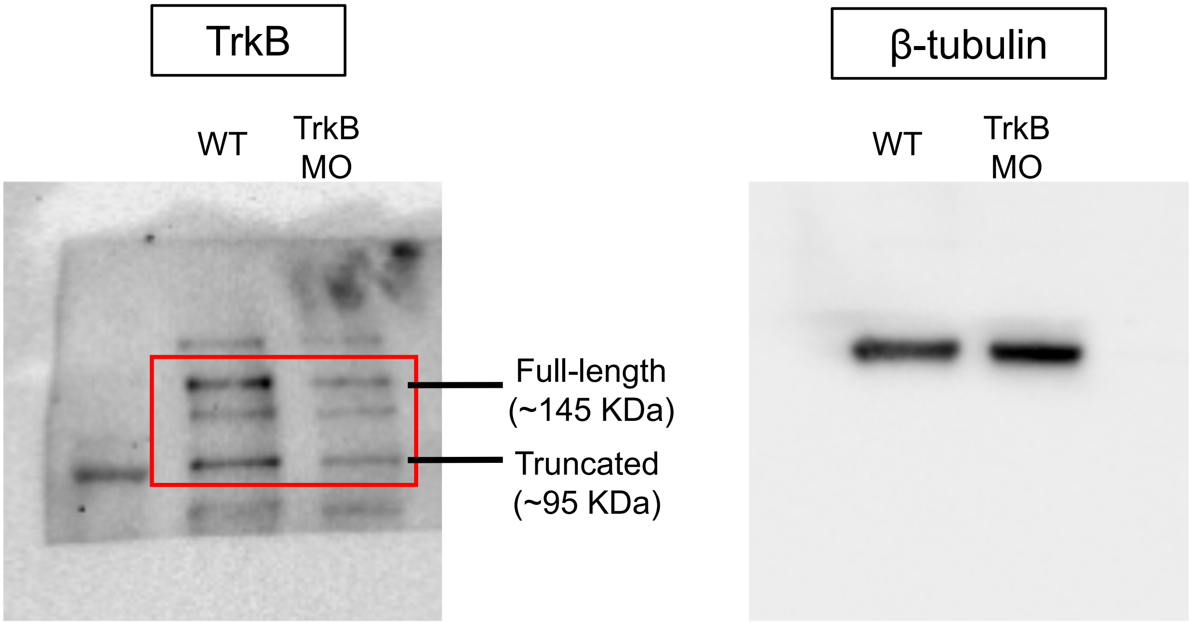

Full-length Western blot in Fig. S7a

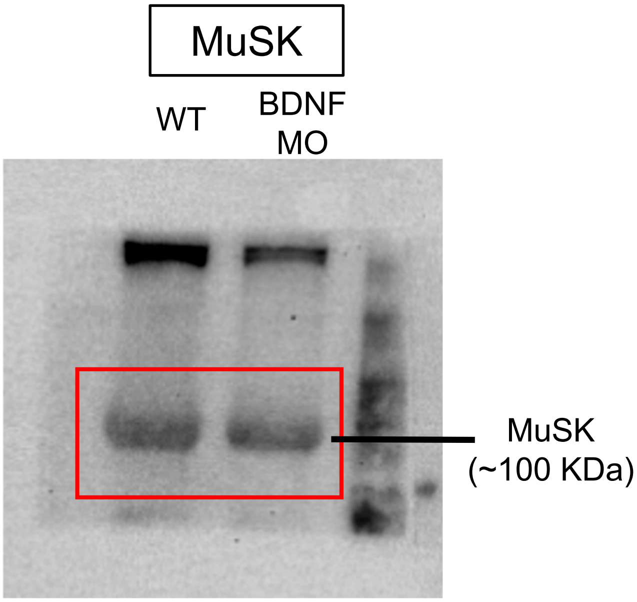

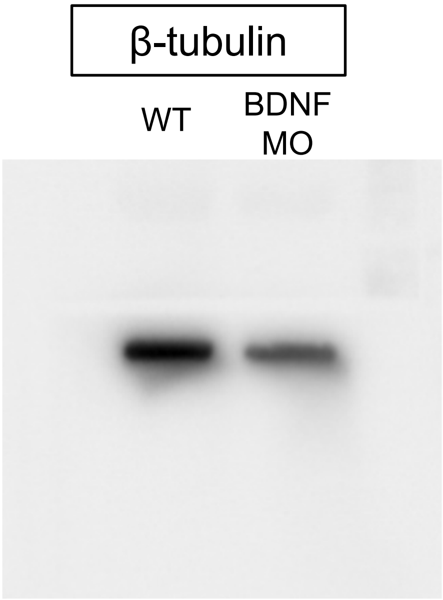

Supplement: Supplementary file 2 — Original data [file 41418_2024_1404_MOESM2_ESM.pdf]
